# Phosphorylation of INF2 by AMPK promotes mitochondrial fission and oncogenic function in endometrial cancer

**DOI:** 10.1038/s41419-024-06431-0

**Published:** 2024-01-17

**Authors:** Yan Ding, Zeheng Lv, Wenxin Cao, Wenming Shi, Qizhi He, Kun Gao

**Affiliations:** 1 Department of Clinical Laboratory, Shanghai First Maternity and Infant Hospital, School of Medicine, Tongji University, Shanghai, 200092 China; 2 Shanghai Key Laboratory of Maternal Fetal Medicine, Shanghai Institute of Maternal-Fetal Medicine and Gynecologic Oncology, Shanghai First Maternity and Infant Hospital, School of Medicine, Tongji University, Shanghai, 200092 China; 3 School of Public Health, Li Ka Shing Faculty of Medicine, The University of Hong Kong, Hong Kong, 999077 China; 4 Department of Pathology, Shanghai First Maternity and Infant Hospital, School of Medicine, Tongji University, Shanghai, PR China

**Keywords:** Endometrial cancer, Mitochondria

## Abstract

Mitochondria are highly dynamic organelles capable of altering their sizes and shapes to maintain metabolic balance through coordinated fission and fusion processes. In various cancer types, mitochondrial hyperfragmentation has been frequently observed, contributing to the progression of cancer toward metastasis. Inverted formin 2 (INF2), which resides in the endoplasmic reticulum (ER), has been found to accelerate actin polymerization and drive mitochondrial fission. In this study, we demonstrate that INF2 expression is significantly upregulated in endometrial cancer (EC) and is associated with a poor prognosis in EC patients. INF2 promotes anchorage-dependent and independent EC cell growth in part by facilitating mitochondrial fission. Furthermore, in conditions of energy stress, AMP-activated protein kinase (AMPK) phosphorylates INF2 at Ser1077, leading to increased localization of INF2 to the ER and enhanced recruitment of the dynamin-related protein 1 (DRP1) to mitochondria. This AMPK-mediated phosphorylation of INF2 at Ser1077 facilitates mitochondrial division and promotes EC cell growth. Pathological examination using immunohistochemical analyses revealed a positive correlation between AMPK activity and phosphorylated INF2 (Ser1077) in EC specimens. Collectively, our findings uncover novel molecular mechanisms involving the AMPK-INF2 axis, which regulates mitochondrial dynamics and malignant cell growth in EC.

## Introduction

Endometrial cancer (EC), the most prevalent cancer affecting the uterine lining in the US, poses a significant threat to women’s health. With 65,950 new cases and 12,550 deaths reported, it is alarming that the mortality rate of EC is increasing, unlike most cancers [1]. EC is categorized into two types: type I is estrogen-dependent, mainly including grades I and II endometrioid adenocarcinoma and estrogen-progesterone receptor-positive endometrial cancer, with a 5-year overall survival (OS) of 85%; type II is estrogen-independent, and the pathological types are grade III endometrioid carcinoma, serous carcinoma, clear cell carcinoma, undifferentiated carcinoma and carcinosarcoma, with a 5-year OS of less than 55% [2]. Various high-risk factors are associated with EC, including continuous estrogen exposure, metabolic syndrome, advanced age, early menarche, delayed menopause, family history, and genetic susceptibility [2, 3]. However, the exact pathogenesis of the disease remains unclear, and there have been no significant advancements in the treatment of advanced and recurrent endometrial cancer. Therefore, it is imperative to explore new molecular mechanisms and therapeutic targets that are linked to the development and occurrence of EC.

Tumors frequently experience energy stress, and mitochondria play a critical role in supporting cell survival under such conditions. Mitochondria are dynamic organelles engaged in a continuous balancing act between division and fusion, collectively known as mitochondrial dynamics [4]. The regulation of these dynamics has significant implications for various aspects of tumor biology, including cell proliferation [5], cell migration [6], tumor metabolism [7], and the maintenance of tumor stem cell characteristics [8]. It is now widely accepted that mitochondrial fission promotes tumor formation, whereas mitochondrial fusion acts as a suppressor of tumorigenesis [9]. Numerous studies have demonstrated that an imbalance in mitochondrial dynamics, leading to mitochondrial fragmentation, is associated with tumor development in various cancers such as lung cancer [10], breast cancer [6], thyroid cancer [11], medulloblastoma [12], liver cancer [13], and EC [14, 15].

AMP-activated protein kinase (AMPK) serves as a vital energy sensor within cells, becoming activated when encountering energy stress. Its activation triggers a series of molecular events aimed at restoring metabolic equilibrium by shifting metabolism from an anabolic to a catabolic state [16]. Recent research has demonstrated that AMPK also plays a pivotal role in promoting mitochondrial division through the phosphorylation of specific downstream substrates [17, 18]. Notably, AMPK activation leads to phosphorylation of the outer mitochondrial membrane protein (MFF), resulting in increased recruitment of the dynamin-related protein 1 (DRP1) to the mitochondria and subsequent facilitation of mitochondrial fission [17, 18]. The activation of AMPK triggers a cascade of molecular events that culminate in the division of mitochondria, promoting cellular energy balance and adaptation. This process is crucial for maintaining mitochondrial quality control, cellular metabolism, and overall cellular health.

Inverted formin 2 (INF2) is a vertebrate-specific protein that plays a crucial role in nucleating the microfilament cytoskeleton and facilitating mitochondrial division [19, 20]. It polymerizes actin in a myosin II-dependent manner at the endoplasmic reticulum (ER), enhancing ER-mitochondria contacts. This actin polymerization recruits DRP1, leading to the formation of a contraction ring and subsequent mitochondrial outer membrane (OMM) contraction [21, 22]. Mutations in the INF2 gene are associated with focal segmental glomerular sclerosis (FSGS) [23] and Charcot-Marie-Tooth disease (CMTD) [24]. Additionally, INF2 dysregulation is implicated in tumor progression and metastasis, with elevated expression observed in cancers such as glioblastoma [25], triple-negative breast cancer [26], and gastric cancer [27]. This overexpression promotes cellular morphological changes and invasive properties.

In this study, we reveal that INF2 expression is upregulated in EC, and its high expression predicts a poor prognosis. Depletion of INF2 inhibits the mitochondrial division of EC cells. Moreover, phosphorylation of INF2 at Ser1077 is induced by AMPK activation, which leads to enhanced mitochondrial fission and EC cell proliferation. Finally, we establish a positive correlation between AMPK activation and INF2 S1077 phosphorylation at the histopathological level, shedding light on how AMPK regulates mitochondrial dynamics through INF2 phosphorylation.

## Results

### INF2 expression is upregulated in EC and correlates with a poor prognosis in EC patients

**Fig. 1:**
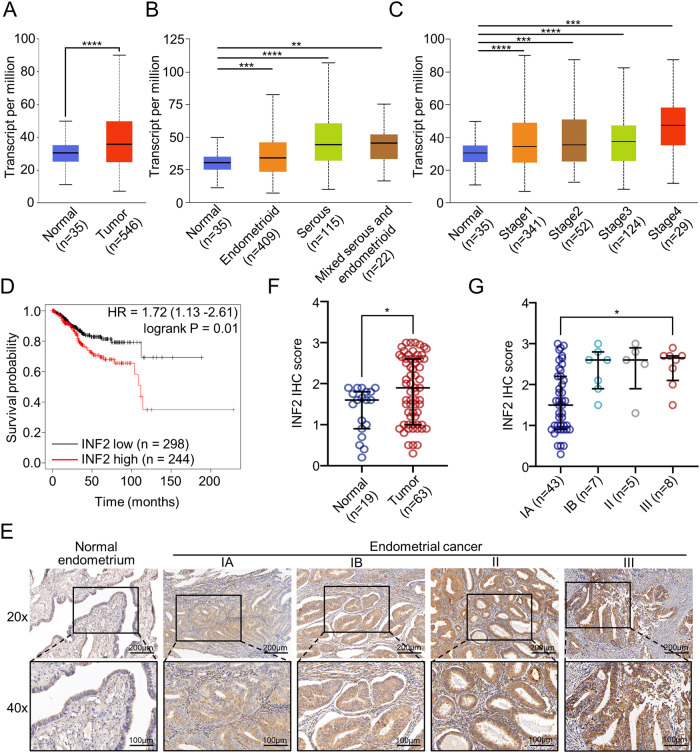
INF2 expression is significantly up-regulated in EC and is associated with the clinical stage and unfavorable prognosis of patients with EC. **A** INF2 mRNA expression in normal endometrium tissues and tumor tissues of EC from TCGA EC cohort (http://ualcan.path.uab.edu/). **B** INF2 mRNA expression in different histological subtypes of EC from TCGA EC cohort. **C** INF2 mRNA expression in EC at different pathological stages from TCGA EC cohort. **D** Kaplan–Meier survival plots of OS according to INF2 mRNA expression in EC patients from the TCGA EC cohort. **E** Representative IHC images of INF2 staining in normal endometrium and EC tissues. **F** INF2 protein expression in normal endometrium tissues and EC tissues from our sample cohort. Data are medians ± interquartile range. **G** INF2 protein expression in EC at different FIGO stages from our sample cohort. Data are medians ± interquartile range. *P* values are calculated using the Student’s t-test in (**A**), the Kruskal–Wallis test in (**B**, **C**, **G**), and the Mann–Whitney test in (**F**). *p < 0.05, **p < 0.01, ***p < 0.001, ****p < 0.0001.

To investigate potential alterations in INF2 expression levels in human cancers, we analyzed the publicly available TCGA dataset to evaluate the mRNA expression pattern of INF2 in human cancer specimens. We found that INF2 mRNA expression was significantly upregulated in various cancer types, including EC when compared to corresponding normal tissues (Supplementary Fig. 1A, Fig. 1A). We further observed that the mRNA expression levels of INF2 were higher in serous and mixed serous/endometrioid EC subtypes (more aggressive) than in endometrioid EC subtypes (less aggressive) (Fig. 1B). Analysis of the TCGA EC dataset revealed that INF2 mRNA expression levels were positively correlated with tumor stages (Fig. 1C). Furthermore, the survival analysis indicated a significant correlation between high INF2 expression and shorter overall survival in the TCGA EC cohort (Fig. 1D). After validating the antibody specificity for Immunohistochemistry (IHC) analysis in parental and INF2 KO HEC-1B cells (Supplementary Fig. 1B), we evaluated INF2 protein expression in EC through IHC analysis of a tissue microarray comprised of 63 EC and 19 adjacent normal tissues from our sample cohort (Supplementary Table 1). Our results showed a substantial upregulation of INF2 protein in EC tissues compared to adjacent normal tissues (Fig. 1E, F), and that INF2 expression increased with FIGO tumor stage (IA to III) (Fig. 1E, G). Taken together, the analysis of the TCGA cohort and our sample cohort consistently suggest that INF2 expression is aberrantly increased in EC, and is correlated with advanced tumor stage and prognosis in EC patients.

### INF2 promotes EC cell proliferation partly by regulating mitochondrial dynamics

**Fig. 2:**
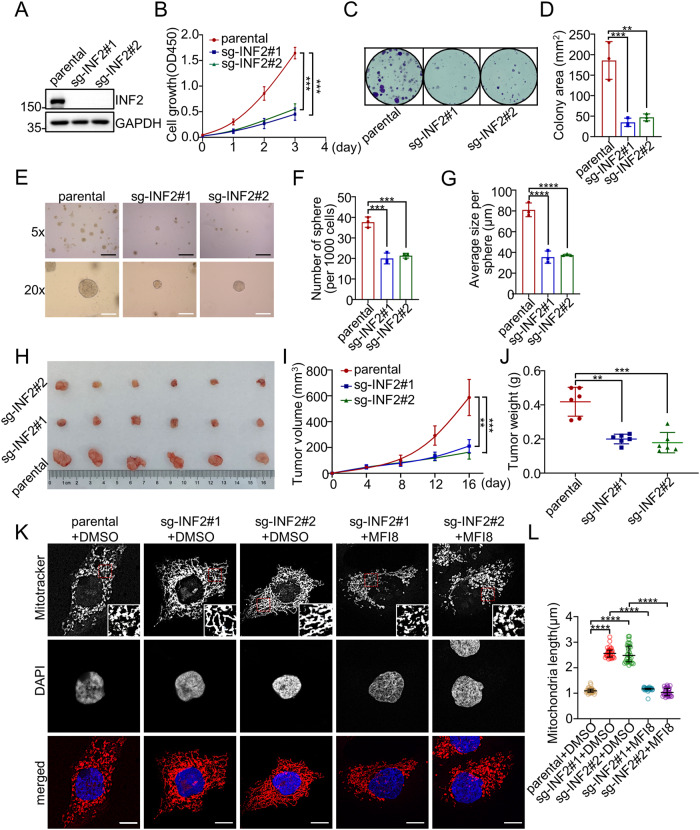
INF2 promotes EC cell proliferation partly by regulating mitochondrial dynamics. **A** INF2 KO HEC-1B cells were generated through CRISPR/Cas 9 gene editing methods. The WCLs from parental and INF2 KO HEC-1B cells were prepared for WB with the indicated antibodies. **B** CCK-8 assays were performed in parental and INF2-KO HEC-1B cells. Data are shown as means ± SD (*n* = 3). **C**, **D** Colony formation assays were performed in parental and INF2-KO HEC-1B cells. The area of clones in (**C**) was analyzed statistically and shown in (**D**). Data are shown as means ± SD (*n* = 3). **E–G** 3D sphere formation assays were performed in parental and INF2-KO HEC-1B cells, scale bar: 5×, 200 μm; 20 ×, 50 μm. The numbers and sizes of cell spheres were analyzed statistically and shown in (**F**, **G**). Data are shown as means ± SD (*n* = 3). **H–J** Xenograft tumor assays were performed to detect the growth ability of parental and INF2-KO HEC-1B cells in vivo. Tumor growth was measured every four days for 16 days. Tumors in each group at day 16 were harvested and photographed (**H**), and tumor volume (**I**) at each time point and tumor weight (**J**) were documented. Data are shown as means ± SD (*n* = 6). **K**, **L** Parental and INF2-KO HEC-1B cells were treated with DMSO or MFI8 (20 μM) for 6 h, then the cells were stained with DAPI and Mitotracker Orange. Representative confocal images are shown. Scale bar: 10 μm. The mitochondrial lengths were analyzed statistically and shown in (**L**). Data are shown as medians ± interquartile range (*n* = 30). *P* values are calculated using the Two-way ANOVA test in (**B**, **I**), the One-way ANOVA test in (**D**, **F**, **G**), the Brown-Forsythe ANOVA test in (**J**), and the Kruskal–Wallis test in (**L**). **p < 0.01, ***p < 0.001, ****p < 0.0001.

To investigate the role of INF2 in EC pathology, we initially examined its expression in multiple EC cell lines. Our findings revealed that INF2 was abundantly expressed in most EC cell lines, except for SPEC-2 and AN3CA cells (Supplementary Fig. 2A). To further explore the functional significance of INF2 in EC, we used CRISPR/Cas9-based genome editing methods to generate INF2 KO HEC-1B cells with two different single guide RNAs (sgRNAs) (Supplementary Fig. 3A-D). Subsequently, WB analysis was performed, which demonstrated that INF2 protein was totally ablated in the two cell clones (Fig. 2A). We observed that INF2 KO significantly reduced the growth of HEC-1B cells in vitro, as evidenced by CCK-8 (Fig. 2B), colony formation (Fig. 2C, D), and EdU incorporation assays (Supplementary Fig. 2B, C). Furthermore, INF2 KO HEC-1B cells showed a marked reduction in the number and size of 3D spheres compared to parental cells, indicating that INF2 KO suppresses the anchorage-independent growth of EC cells (Fig. 2E-G). Additionally, subcutaneous inoculation of parental and INF2 KO HEC-1B cells in nude mice showed that INF2 KO significantly attenuated HEC-1B xenograft tumor growth (Fig. 2H-J). Taken together, these results indicate that INF2 plays a pivotal role in promoting the malignant proliferative potential of endometrial cancer, as also verified in another EC cell line, Ishikawa cells (Supplementary Fig. 2D–L).

Previous studies have established that INF2-mediated actin polymerization is a crucial initial step in mitochondrial fission. Consistently, our study revealed a significant increase in mitochondrial length in INF2 KO HEC-1B or Ishikawa cells, along with fusion into tube-like structures and reduced division (Fig. 2K, L, Supplementary Fig. 2M, N), confirming that INF2 controls mitochondrial dynamics in EC cells. To investigate whether INF2-mediated mitochondrial fission is required for the malignant proliferative potential of EC cells, we treated INF2 KO HEC-1B or Ishikawa cells with the mitochondrial fusion inhibitor MFI8 [28]. Remarkably, MFI8 treatment reversed the INF2 KO-induced reduction of mitochondrial length and partially rescued the proliferation inhibition caused by INF2 KO (Fig. 2K, L, Supplementary Fig. 2B, C). Taken together, these results indicate that INF2 promotes EC cell proliferation partly by regulating mitochondrial dynamics.

### AMPK interacts with INF2 and phosphorylates INF2 at Ser1077

**Fig. 3:**
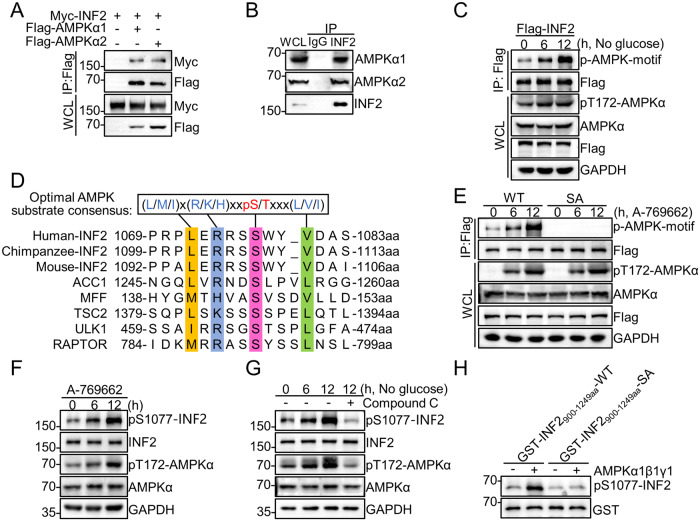
AMPK interacts with INF2 and phosphorylates INF2 at Ser1077. **A** WB analysis of the indicated proteins in WCLs and co-IP samples of anti-FLAG antibody obtained from 293T cells transfected with the indicated plasmids. **B** Co-IP using anti-INF2 antibody in WCLs prepared from HEC-1B cells, followed by WB analysis with the indicated antibodies. **C** WB analysis of the indicated proteins in WCLs and co-IP samples of anti-FLAG antibody obtained from 293 T cells transfected with the indicated plasmids and treated with glucose deprivation for the indicated times. **D** Amino acid sequence alignment of putative AMPK phosphorylation sites in INF2 and other known AMPK substrates containing AMPK substrate consensus motif. **E** WB analysis of the indicated proteins in WCLs and co-IP samples of anti-FLAG antibody obtained from 293 T cells transfected with the indicated plasmids and treated with A-769662 (100 μM) for the indicated times. **F** WB analysis of the indicated proteins in WCLs from HEC-1B cells treated with A-769662 (100 μM) for the indicated times. **G** WB analysis of the indicated proteins in WCLs from HEC-1B cells treated with glucose deprivation at the indicated times. The cells were pretreated with Compound C 2 h prior to glucose deprivation. **H** Recombinant GST-INF2_900-1249aa_-WT or GST-INF2_900-1249aa_-S1077A segments were subjected to phosphorylation by recombinant AMPKα1/β1/γ1 as detected using in vitro kinase assays. The reaction products were separated by SDS-PAGE and the phosphorylation status was detected using an anti-INF2 (phospho-Ser1077) antibody.

While the essential roles of INF2 in mitochondrial dynamics are well-established, the influence of upstream signals or physiological conditions on this process remains poorly understood. AMPK, a sensor for nutrients and energy, integrates cellular energetics to regulate mitochondrial dynamics and maintain energetic homeostasis [29]. we aimed to investigate whether AMPK acts as an upstream regulator of INF2. We demonstrated that there was an interaction between ectopically overexpressed INF2 and either AMPKα1 or α2 (Fig. 3A). Furthermore, by using endogenous antibodies to precipitate INF2 protein from HEC-1B and Ishikawa endometrial cancer cells, we confirmed the presence of endogenous AMPKα1/α2 protein (Fig. 3B, Supplementary Fig. 4A).

As AMPK functions as a serine/threonine protein kinase, we proceeded to investigate whether INF2 is a phosphorylation substrate of AMPK. Glucose deprivation can activate AMPK through both AMP-dependent and AMP-independent mechanisms [30, 31]. To assess the changes in INF2 phosphorylation levels under AMPK activation induced by glucose deprivation, we employed a phospho-AMPK-motif antibody. Our results revealed a gradual increase in the phosphorylation level of INF2 in a time-dependent manner (Fig. 3C). Analysis of high-throughput phosphoproteomic data from the Phosphosite database (https://www.phosphosite.org/) unveiled a potential AMPK phosphorylation site on INF2 at Ser1077. This evolutionarily conserved site (LERRSpSWYV) closely resembles the canonical AMPK substrate motif (LxRxxpSxxxV) (Fig. 3D).

To delve deeper into this observation, we generated a phosphorylation-null mutant of INF2 at Ser1077 (designated as S1077A). Subsequently, we transfected Flag-INF2-WT or S1077A plasmids into 293T cells and treated them with the allosteric AMPK activator A-769662. We found that AMPK activation progressively enhanced the p-AMPK-motif signal of INF2 in a time-dependent manner, while no such signal was detected in the INF2-S1077A mutant (Fig. 3E). Moreover, we developed a phosphorylation-specific antibody targeting INF2 Ser1077 (Supplementary Fig. 4B) and observed that AMPK activation by A-769662 or glucose deprivation markedly promoted the INF2 (phospho-Ser1077) signal (Fig. 3F, G, Supplementary Fig. 4C, D). Furthermore, treatment with the AMPK inhibitor Compound C impeded the increase in the INF2 (phospho-Ser1077) signal induced by glucose starvation (Fig. 3G, Supplementary Fig. 4D).

The results obtained above strongly suggest the phosphorylation of INF2 Ser1077 in response to AMPK activation in EC cells. To directly investigate whether AMPK phosphorylates INF2, we performed in vitro kinase assays using a recombinant INF2_900-1249aa_ segment that contains Ser1077 as a substrate. Our results demonstrated that the WT INF2_900-1249a_ segment, but not the S1077A mutant, was efficiently phosphorylated by the purified AMPK kinase complex, as confirmed by the INF2 (phospho-Ser1077) antibody (Fig. 3H). Taken together, these results provide compelling evidence that AMPK can directly phosphorylate INF2 at Ser1077 in response to cellular energy stress.

### AMPK promotes mitochondrial fission partly via INF2

**Fig. 4:**
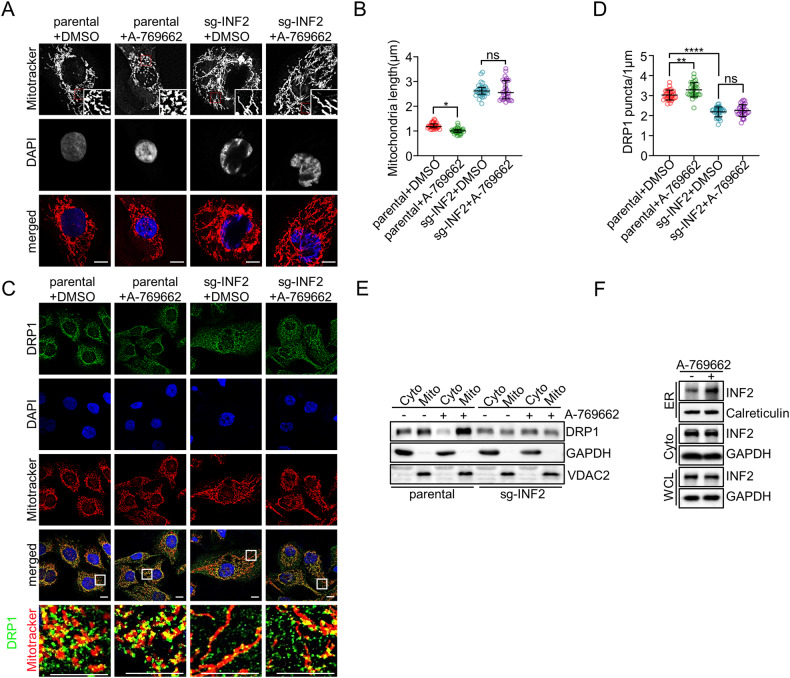
AMPK activation promotes mitochondrial fission via INF2. **A**, **B** Parental and INF2-KO HEC-1B cells were treated with DMSO or A-769662 (100 μM) for 6 h, then the cells were stained with DAPI and Mitotracker Orange. Representative confocal images are shown. Scale bar: 10 μm. The mitochondrial lengths were analyzed statistically and shown in (**B**). Data are shown as means ± SD (*n* = 30). **C**, **D** Parental and INF2-KO HEC-1B cells were treated with DMSO or A-769662 (100 μM) for 6 h, then the cells were stained with DAPI, DRP1, and Mitotracker Orange. Representative confocal images are shown (**C**). Scale bar: 10 μm. Quantification of DRP1 puncta per mitochondrial length in (**D**). Data are medians ± interquartile range (*n* = 30). **E** Parental and INF2-KO HEC-1B cells were treated with DMSO or A-769662 (100 μM) for 6 h. The cytosol and purified mitochondrial Fractions were isolated and detected by WB analysis with the indicated antibodies. **F** HEC-1B cells were treated with DMSO or A-769662 (100 μM) for 6 h. The ER Fractions were isolated and detected by WB analysis with the indicated antibodies. *P* values are calculated using the One-way ANOVA test in (**B**), and the Kruskal–Wallis test in (**D**). *p < 0.05, **p < 0.01, ****p < 0.0001, ns: no significant.

We aimed to investigate whether the phosphorylation of INF2 induced by AMPK activation has an impact on mitochondrial fission in EC cells. Our results demonstrate that treatment with A-769662 significantly reduces the length of mitochondria in parental HEC-1B or Ishikawa cells, but this effect is not observed in INF2-KO cells (Fig. 4A, B, Supplementary Fig. 4E, F). DRP1 recruitment to mitochondria is a crucial downstream event in INF2-mediated mitochondrial fission. Immunofluorescence analysis (IF) reveals that AMPK activation promotes the formation of DRP1 puncta in HEC-1B cells, whereas no such effect is observed in INF2-KO HEC-1B cells (Fig. 4C, D). To further confirm these findings, we isolated mitochondrial and cytoplasmic proteins from cells treated with dimethyl sulfoxide (DMSO) or A-769662. Consistently, an increase in DRP1 puncta on mitochondria is observed after AMPK activation, while no such effect is observed in INF2-KO cells (Fig. 4E). However, AMPK activation did not exert any discernible impact on the interaction between INF2 and DRP1 (Supplementary Fig. 4G).

It is known that the subcellular localization of INF2 can be dynamically regulated. Previous research demonstrated that the E3 ubiquitin ligase, speckle-type POZ protein (SPOP), triggers atypical polyubiquitination of INF2, reducing its localization in the ER and subsequently impairing its ability to facilitate mitochondrial fission [32]. With this knowledge, we hypothesized that AMPK activation might alter the subcellular localization of INF2. To test this hypothesis, we conducted ER fractionation experiments and found that the ER localization of INF2 increases after AMPK activation (Fig. 4F). We also showed that AMPK activation did not exert any discernible impact on the SPOP-mediated INF2 ubiquitination. (Supplementary Fig. 4H). Taken together, our results indicate that INF2 plays a critical role in AMPK activation-promoted mitochondrial fission.

### Phosphorylation of INF2 at Ser1077 enhances mitochondrial fission and EC cell growth

**Fig. 5:**
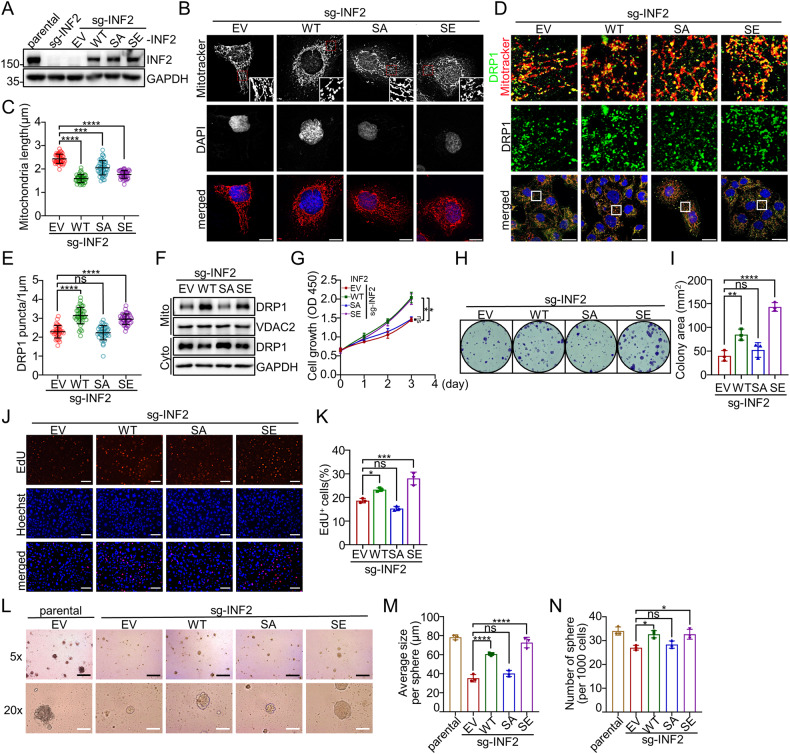
AMPK-mediated phosphorylation of INF2 at Ser1077 promotes EC cell proliferation. **A** WB analysis of the indicated proteins in WCLs from parental or INF2-KO HEC-1B cells stably expressing EV, INF2-WT, SA, or SE mutant. **B**, **C** INF2-KO HEC-1B cells stably expressing EV, INF2-WT, SA, or SE mutant were stained with DAPI and Mitotracker Orange. Representative confocal images are shown in (**B**). Scale bar: 10 μm. The mitochondrial lengths were analyzed statistically and shown in (**C**). Data are medians ± interquartile range (*n* = 30). **D**, **E** INF2-KO HEC-1B cells stably expressing EV, INF2-WT, SA, or SE mutant were stained with DAPI, DRP1, and Mitotracker Orange. Representative confocal images are shown (**D**). Scale bar: 10 μm. Quantification of DRP1 puncta per mitochondrial length in (**E**). Data are shown as means ± SD (*n* = 30). **F** The cytosol and purified mitochondrial fractions of INF2-KO HEC-1B cells stably expressing EV, INF2-WT, SA, or SE mutant were isolated and detected by WB analysis with the indicated antibodies. (**G**) CCK-8 assays were performed in parental and INF2-KO HEC-1B cells. Data are shown as means ± SD (n = 3). (**H**, **I**) Colony formation assays were performed in INF2-KO HEC-1B cells stably expressing EV, INF2-WT, SA, or SE mutant. The area of clones in (**H**) was analyzed statistically and shown in (**I**). Data are shown as means ± SD (*n* = 3). (**J**, **K**) EdU assays were performed in INF2-KO HEC-1B cells stably expressing EV, INF2-WT, SA, or SE mutant. The proportion of EdU^+^ positive cells was analyzed statistically and shown in (**K**). Data are shown as means ± SD (*n* = 30). **L–N** 3D sphere formation assays were performed using parental and INF2-KO HEC-1B cells stably expressing EV, INF2-WT, SA, or SE mutant, scale bar: 5×, 200 μm; 20×, 50 μm. The numbers and sizes of cell spheres were analyzed statistically and shown in (**M**, **N**). Data are shown as means ± SD (*n* = 3). *P* values are calculated using the Kruskal-Wallis test in (**C**), the one-way ANOVA test in (**E**, **I**, **K**, **M**, **N**), and the Two-way ANOVA test in (**G**). *p < 0.05, **p < 0.01, ***p < 0.001, ****p < 0.0001, ns no significant.

To elucidate the biological significance of INF2 phosphorylation mediated by AMPK, we introduced INF2-WT, phosphorylation-deficient S1077A, or phospho-mimicking S1077E mutant into INF2-KO HEC-1B cells. These cell lines were designated as WT, SA, or SE, respectively (Fig. 5A, Supplementary Fig. 5A, B). By measuring mitochondrial length, we observed that the reintroduction of INF2-WT or SE mutant largely reversed the increase in mitochondrial fusion induced by INF2 KO. However, no such effect was observed in cells reintroduced with the INF2-SA mutant (Fig. 5B, C). We performed the same procedures in Ishikawa cells and obtained similar results (Supplementary Fig. 5C, D). Subsequently, we conducted IF experiments to evaluate DRP1 puncta formation in these cells. We found that the reintroduction of INF2-WT or SE mutants largely reversed the decrease in DRP1 puncta formation induced by INF2 KO. Conversely, no such effect was observed in cells reintroduced with the INF2-SA mutant (Fig. 5D, E). Furthermore, we conducted mitochondria fractionation experiments and found that the decrease in mitochondrial localization of DRP1 caused by INF2 KO was largely reversed by the reintroduction of INF2-WT or SE mutants. However, the SA mutant failed to restore mitochondrial DRP1 localization (Fig. 5F). These findings indicate that INF2 phosphorylation at Ser1077 promotes the accumulation of DRP1 on mitochondria, thereby mediating mitochondrial fission.

To further investigate the relationship between INF2 phosphorylation at Ser1077 and EC cell proliferation, we conducted CCK-8 assays (Fig. 5G), colony formation assays (Fig. 5H, I), and EdU assays (Fig. 5J, K). Collectively, these experiments confirmed that INF2 phosphorylation at Ser1077 plays a positive role in promoting EC cell growth. Additionally, 3D sphere formation assays demonstrated that phosphorylation of INF2 at Ser1077 enhances anchorage-independent cell growth (Fig. 5L–N). We performed similar experiments in Ishikawa cells and obtained similar results (Supplementary Fig. 5E–L). Taken together, these findings indicate that INF2 phosphorylation at Ser1077 enhances mitochondrial fission and promotes EC cell growth.

### AMPK activation positively correlates with INF2 Ser1077 phosphorylation in EC patients

**Fig. 6:**
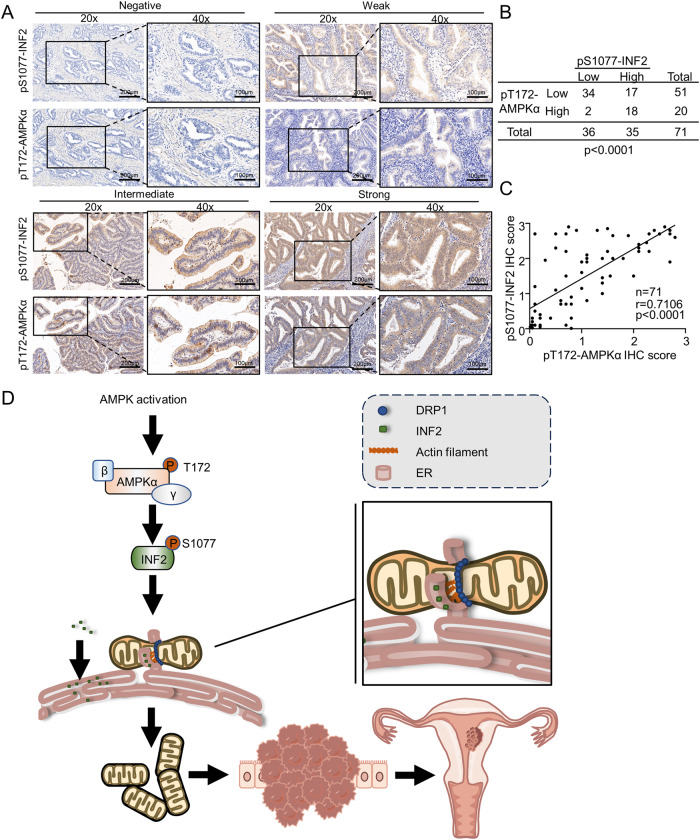
Histopathological verification of the correlation between AMPK activation and INF2 Ser1077 phosphorylation level. **A** Representative images of IHC staining of 71 EC specimens with AMPKα (phospho-Thr172) and INF2 (phospho-Ser1077) antibodies. **B, C** Statistical analysis of the IHC experiments in (**A**). **D** A schematic model summarizing the findings of the present study generated by Figdraw (https://www.figdraw.com/). *P* values are calculated using Fisher’s exact test in (**B**) and Spearman’s rank correlation test in (**C**).

Finally, we investigate whether the regulation of INF2 Ser 1077 phosphorylation by AMPK can be observed in EC specimens. AMPK activation is promoted by the phosphorylation at Thr172 of the AMPKα subunits. Therefore, AMPKα (phospho-Thr172) is widely used as a surrogate marker for AMPK activation. Firstly, we evaluated AMPKα expression in both normal endometrium and EC tissues by IHC analysis. No significant differences in AMPKα intensity were observed between the normal and tumorous samples (Supplementary Fig. 5M, N). Next, using both AMPKα (phospho-Thr172) and INF2 (phospho-Ser1077) antibodies, we observed a statistically significant correlation between AMPKα (phospho-Thr172) and INF2 (phospho-Ser1077) in EC specimens (Fig. 6A–C). Taken together, our results suggest that AMPK plays a role in EC tumorigenesis by promoting INF2 phosphorylation.

## Discussion

In our study, we have established that INF2 acts as an oncogene in EC. Furthermore, we have demonstrated that AMPK is responsible for phosphorylating INF2 at Ser1077. This phosphorylation event leads to the promotion of mitochondrial fission and fragmentation. Specifically, INF2 is localized to the ER, which subsequently causes the delocalization of DRP1 to the outer mitochondrial membrane. The phosphorylation of INF2 at Ser1077 is closely associated with increased anchorage-dependent and anchorage-independent growth of EC cells. At the histopathological level, there is a positive correlation between AMPK activation and INF2 Ser1077 in EC patients. These findings reveal novel molecular mechanisms related to the AMPK-INF2 axis, which plays a critical role in regulating mitochondrial dynamics and fostering malignant cell growth in EC.

Existing studies on post-translational modifications of INF2 primarily focus on prenylation and ubiquitination. These modifications primarily affect mitochondrial fission by regulating the localization of INF2 in the ER. Specifically, the C-terminal prenylation of INF2 facilitates its tight binding to the ER and correct positioning [21, 33]. On the other hand, the atypical ubiquitination of INF2 by the CRL3^SPOP^ ubiquitin ligase complex reduces its ER localization and inhibits mitochondrial division [32]. Moreover, a recent study has identified that F-box protein 7 (FBXO7) induces ubiquitination and degradation of INF2, thereby inhibiting INF2-DRP1 axis-associated mitochondrial fission [15]. Our findings contribute to the understanding of INF2 post-translational modifications, demonstrating that increased phosphorylation of INF2 Ser1077 in EC cells enhances ER localization, leading to greater recruitment of DRP1 to mitochondria.

Previous research has indicated that the process of mitochondrial division initiates at the point where mitochondria make contact with the ER [21]. Initially, mitochondrial DNA replication occurs at this contact site, and the presence of INF2 on the ER triggers actin polymerization. This actin polymerization strengthens the connection between the ER and mitochondria in a manner dependent on myosin II [22]. Simultaneously, the increased interaction between the endoplasmic reticulum and mitochondria facilitates the transfer of more calcium from the ER to the mitochondria. This results in the pre-contraction of both the OMM and IMM. Actin filaments subsequently recruit DRP1 to the OMM for further assembly into complete rings, leading to OMM contraction [22]. Our study adds to the understanding of how post-translational modification of INF2 regulates mitochondrial dynamics by influencing the localization of DRP1. Specifically, the phosphorylation of INF2 S1077 promotes more localization of INF2 on the ER, which, in turn, leads to increased DRP1 recruitment.

Under conditions of energy stress, there is an increase in the AMP/ATP ratio, which subsequently activates AMPK. AMPK activation plays a crucial role in restoring cellular energy levels by phosphorylating multiple downstream substrates, thereby balancing cell metabolism [34]. The regulation of energy metabolism by AMPK is closely intertwined with mitochondrial function [29]. Inhibitors of the electron transport chain induce AMPK-dependent mitochondrial division, and treatment with small molecule activators of AMPK directly triggers mitochondrial division [17]. AMPK regulates the dynamic changes of mitochondria during energy stress through the MFF-DRP1 axis [17]. Specifically, phosphorylation of activated AMPK modifies more MFF located in the outer membrane of mitochondria, leading to the recruitment of more DRP1 to mitochondria and promoting mitochondrial division [17]. Additionally, quantitative phosphoproteomics comparing wild-type and AMPKα1/α2 double KO cells have identified ARMC10 as a substrate of AMPK phosphorylation [35]. AMPK phosphorylation modifies ARMC10 Ser45 to promote mitochondrial division [35]. Overexpression of ARMC10 is sufficient to promote the process of mitochondrial division, and further studies have found that AMPK phosphorylation of ARMC10 may be achieved through the interaction of its mitochondrial division-related protein MFF with mitochondrial fission 1 protein (Fis1) [35]. These studies, together with ours, indicated that AMPK simultaneously phosphorylates multiple substrates to modulate mitochondrial dynamics.

Targeting mitochondrial dynamics shows promise as a treatment strategy to inhibit tumorigenesis and development by restoring cell function. Several small molecules targeting key proteins involved in mitochondrial dynamics have been used in trials [36]. Mdivi-1, which was first identified in 2008, inhibits DRP1 assembly and GTPase activity, thereby inhibiting mitochondrial fission [37]. Mdivi-1 has demonstrated the ability to inhibit the proliferation and migration of EC cells, possibly by regulating reactive oxygen species in mitochondria [15]. Given that high INF2 expression enhances EC proliferation by promoting mitochondrial division, inhibition of mitochondrial division may be a viable approach for the treatment of EC. In conclusion, our findings offer potential value for the development of molecular targeted therapies focused on mitochondrial fission-related factors in the future. Additionally, our study provides theoretical insights into the role of AMPK in mitochondrial dynamics and cancer development.

## Materials and methods

### Cell culture, transfection, and lentiviral infection

The 293T, Ishikawa, and HEC-1B cell lines were obtained from the Chinese Academy of Science Committee Type Culture Collection cell bank (Shanghai, China). The 293T cells were cultured in Dulbecco’s Modified Eagle Medium (DMEM) supplemented with 10% fetal bovine serum (FBS) and 1% penicillin/streptomycin, while Ishikawa and HEC-1B cells were maintained in DMEM/F12 supplemented with 10% FBS and 1% penicillin/streptomycin. All cells were cultured at 37 °C with 5% CO_2_. For glucose deprivation treatment, the cells were rinsed twice and then incubated in glucose-free DMEM/F12 medium (Ishikawa and HEC-1B) or DMEM medium (293T), both supplemented with 10% FBS and 1% penicillin/streptomycin. Additionally, 1 mM sodium pyruvate was added to the DMEM medium. For lentiviral transfection, pCDH overexpression plasmids and virus-packing constructs were transfected into 293T cells. The viral supernatant was collected after 48 h and concentrated using the Lenti-X concentrator kit (Clontech) at 4 °C overnight. Thereafter, Ishikawa and HEC-1B cells were infected with the concentrated viral supernatant in the presence of HistransG A (25×) to facilitate lentiviral transduction. EZ trans reagent was used for transient transfection.

### Antibodies, recombinant proteins, and chemicals

Detailed information regarding the antibodies, recombinant proteins, and chemicals utilized in this study are listed in Supplementary Table 2.

### CRISPR/Cas9-mediated INF2 KO cells generation

The INF2 gene was targeted using single guide RNAs (sgRNAs) designed with an online CRISPR design tool (http://crispr.mit.edu/). Supplementary Table 3 provides the sequences of the sgRNAs. Oligonucleotides were synthesized, annealed, and ligated into the pX459 construct from Dr. Feng Zhang’s lab (Addgene) linearized with BbsI. Cells were seeded into 6-well plates one day prior to transfection, and plasmid transfections were carried out using PEI. After 24–48 h, cells were selected with puromycin (2 μg/ml for Ishikawa and 1 μg/ml for HEC-1B) for 3–4 days. Surviving cells were then seeded in a 96-well plate with 100 cells per well. After 2–3 weeks, single-clone colonies were picked and screened using western blot (WB) analysis. Positive clones were subsequently validated through Sanger sequencing.

### Immunofluorescence

Cells were plated into a confocal dish at a density of 1 × 10^5^ cells per well. Once the cells had adhered, they were rinsed twice with PBS and fixed with 4% paraformaldehyde (PFA) for 20 min at room temperature (RT), followed by permeabilization with 0.3% TritonX-100 and blocking with 5% donkey serum for 1 h at RT. The primary antibody was then incubated with the cells overnight at 4 °C. After being washed with PBST buffer, the cells were subjected to a 1 h incubation with a secondary antibody labeled with fluorescence at 37 °C. The cells were then counterstained with DAPI before imaging them with a confocal laser scanning microscope (SP8, Leica), which was equipped with a 63*/1.4NA Oil PSF Objective. Subsequently, the images were subsequently processed using ImageJ software.

### Mitochondrial morphology analysis

To analyze mitochondrial morphology, 5 × 10^4^ cells were seeded in a confocal dish one day prior to staining. The staining was performed under light protection by adding 120 nM of prewarmed MitoTrackerTM Orange CMTMRos in serum-free media at 37 °C for 20 min. After staining, cells were fixed in 4% PFA at 37 °C for 15 min and permeabilized with 0.2% TritonX-100 in PBS at RT for 10 min. Nuclear counterstaining was performed using DAPI. The slides were imaged using a confocal laser scanning microscope with a 63*/1.4NA Oil PSF Objective, and quantitative analyses were conducted using ImageJ software.

### In vitro kinase assays

The active AMPK complex (α1/β1/γ1) was purchased from Carna Biosciences. 10 × kinase assay buffer was purchased from Cell Signaling Technology. For GST-tagged fusion proteins expression, the GST-INF2_900-1249aa_ and GST-INF2_900-1249aa_-S1077A plasmids were transformed into BL21 cells and induced by 1 mM Isopropyl β-D-1-thiogalactopyranoside for 7 h at 30 °C. Subsequently, cells were harvested, lysed ultrasonically, and then purified using GST Agarose (Thermo). The purified proteins were then incubated with the active AMPK complex for 30 min at 30 °C in a 20 μL reaction system consisting of 2 μL 10 × kinase assay buffer, 100 μM ATP, 1 μg substrate, and 0.1 μg kinase. The reaction was terminated by adding an SDS-loading buffer, and all samples were directly subjected to SDS-PAGE.

### Immunohistochemistry

TMA slides consisting of 94 localized EC specimens and 38 adjacent normal tissues were obtained from our hospital. Paraffin-embedded tissues went through sequential treatment with graded alcohol and antigen retrieval using citrate buffer (Biotechwell) in a microwave. Subsequently, the sections were treated with 3% H_2_O_2_ for 25 minutes to suppress endogenous peroxidase activity and then thoroughly washed with phosphate-buffered saline (PBS, pH 7.4) twice. A solution of 5% bovine serum albumin (BSA) was evenly applied to cover the tissue on the slide and was incubated at room temperature for 1 h. Next, the slide was incubated with diluted antibodies overnight at 4 °C. After being washed three times with PBS, the sections were incubated with biotin-labeled goat anti-rabbit IgG and stained via a SABC Kit (Biotechwell). Finally, the slide was counterstained with 0.1% hematoxylin and dehydrated, covered, and visualized under a confocal microscope. Each sample was scored based on the staining intensity (0 = negative staining; 1 = weak staining; 2 = intermediate staining; 3 = strong staining) and the proportion of cells. All IHC data were analyzed by a single qualified pathologist to ensure consistency, and the clinical information of EC specimens is provided in Supplementary Table 1. All experimental protocols (No. KS2281) had pre-approval from the Ethics Review Committee of Shanghai First Maternity and Infant Hospital.

### CCK-8 assays

The proliferation rates of HEC-1B and Ishikawa cells were determined using the Cell Counting Kit 8 (CCK-8) (Beyotime). Briefly, cells were seeded in triplicate at a density of 3 × 10^3^ cells per well in 96-well plates. Over the 0 to 3-day incubation period, 10 μL of CCK-8 reagent and 90 μL of serum-free medium were daily added to each well simultaneously, followed by a 90 min incubation at 37 °C. The absorbance of the samples was measured at 450 nm using a microplate absorbance reader (Bio-Rad).

### Colony formation assays

The cells (Ishikawa: 800 cells/well, HEC-1B: 400 cells/well) were plated at 6-well plates. After culturing for 2 weeks, the cells were fixed with 4% PFA for 20 min at RT and stained with 0.5% crystal violet for 20 min. The plates were gently rinsed with water and air-dried at RT, and the number of colonies was captured and quantified using ImageJ software.

### EdU assays

HEC-1B or Ishikawa cells were seeded in triplicate onto 96-well plates at a density of 1 × 10^4^ cells per well. After 24 h, the cells were fixed with 4% PFA for 20 min at RT. Permeabilization was achieved by incubating the cells with 0.3% TritonX-100 (in PBS) for 10 min at RT. Following that, the cells were blocked with 5% BSA at RT for 1 h, and the EdU assessment was conducted according to the manufacturer’s instructions. DNA synthesis was assessed using Alexa Fluor 555 (Beyotime), and the nuclei were stained with DAPI. The number of EdU^+^ cells was captured using a confocal microscope and quantified using ImageJ software.

### Sphere formation assays

The Matrigel (BD Science) was pre-cooled at 4 °C overnight before the experiment. Cell suspensions containing 1 × 10^3^ cells/well in DMEM/F12 with 10% FBS were mixed with Matrigel at a volume ratio of 1:3. This mixture was then added to 24-well ultra-low attachment plates. After a 10 min incubation at 37 °C, fresh media were added. The floating spheres that formed within 1–2 weeks were captured using a microscope, and their number and size were measured using ImageJ software.

### Xenograft tumor growth assays

All experimental procedures (No. TJBG10022102) were approved in advance by the Ethics Review Committee for Animal Experimentation of Shanghai First Maternity and Infant Hospital. BALB^nude^/c^nude^ female mice, aged 4-5 weeks, were bred and maintained under specific pathogen-free conditions. Subsequently, 1 × 10^7^ parental or INF2 KO HEC-1B cells suspended in 100 μl PBS were subcutaneously injected into female BALB^nude^/c^nude^ mice aged 5–6 weeks. Tumor volumes were measured every 4 days using a digital caliper, starting from the day of injection. Tumor volumes were calculated using the ellipsoid volume formula: *V* = (*L* × *W*^2^)/2, where *L* is the length and W is the width. After 16 days of tumor cell injection, the mice were euthanized, and solid tumors were excised and weighed.

### Isolation of the mitochondria and endoplasmic reticulum

For isolation of the mitochondria fraction, the following procedure was conducted, as previously described [38]. Approximately 1 × 10^7^ cells were treated with 0.25% pancreatin for digestion, followed by three washes with PBS. The cells were then resuspended in 200 μl of a hypotonic buffer containing 140 mM KCl, 10 mM EDTA, 5 mM MgCl2, 20 mM HEPES (pH 7.4), and a protease inhibitor. Subsequently, the cells were gently homogenized by pipetting 25 times using a 0.5 ml glass pipette. The resulting homogenate was centrifuged twice, first at 800 g for 10 minutes to separate the nuclei fractions, then at 12,000 × *g* for 35 min to obtain cytoplasm and mitochondria, respectively. The mitochondria fraction was further washed with a mitochondria washing buffer composed of 800 mM KCl, 10 mM EDTA, 5 mM MgCl_2_, 20 mM HEPES (pH 7.4), and a protease inhibitor, to obtain pure mitochondrial extracts, which were subsequently separated using SDS-PAGE. For extraction of the ER fraction, the ER fraction of 3 × 10^8^ cells was extracted using an ER enrichment Kit (Invent), according to the manufacturer’s instructions. The differential expression of INF2 between cytosolic and ER proteins before and after treatment was analyzed by WB.

### In vivo ubiquitination assays

293 T cells were transfected with HA-ubiquitin and indicated constructs. After 36 h, the cells were incubated with 20 μM MG132 for 8 h and then lysed in 1% SDS buffer (Tris [pH 7.5], 0.5 mM EDTA, 1 mM DTT) and boiled for 10 min. For immunoprecipitation, the cell lysates were diluted 10-fold in Tris-HCL buffer and incubated with anti-Flag M2 agarose beads (Sigma) or IgG-conjugated beads (Sigma) for 4 h at 4 °C. The bound beads are then washed four times with BC100 buffer (20 mM Tris-HCl, pH7.9,100 mM NaCl,0.2 mM EDTA, 20% glycerol) containing 0.2% Triton X-100. The proteins were eluted with FLAG peptide for 2 h at 4 °C. The ubiquitinated form of INF2 was detected by WB using an anti-HA antibody.

### Statistical analysis

All experiments were repeated a minimum of three times. Statistical analysis was performed to determine the significance of differences between the two measurements using the unpaired two-tailed Student’s t-test. For samples with non-normal distributions, the medians of the variables between the two groups were compared using the Mann-Whitney test. Spearman’s Rank Correlation test was conducted for correlation analysis. One-way and two-way ANOVA was employed to test for overall differences between groups. The Kruskal–Wallis test was utilized for multiple comparisons involving non-normally distributed data. Normally distributed data are presented as the mean ± SD, while non-normally distributed data are expressed as the median and interquartile range. For all tests, a p-value of less than 0.05 was considered statistically significant between groups, with *p < 0.05, **p < 0.01, ***p < 0.001, ****p < 0.0001 indicating the level of significance. All data analyses were conducted using GraphPad Prism 9 software.

### Supplementary information

Supplementary Materials

Original Data File

## Data Availability

Detailed information on the clinical information of EC specimens, reagents, sequences of sgRNAs, and constructed recombinant DNA can be found in Supplementary Tables 1–4. For the original data, please contact kungao@tongji.edu.cn.
