# Supplementary material for: Phosphorylation of INF2 by AMPK promotes mitochondrial fission and oncogenic function in endometrial cancer
Source: Cell Death Dis. 2024 Jan 17;15(1):65. doi: 10.1038/s41419-024-06431-0 (PMC10794193; doi:10.1038/s41419-024-06431-0)
Supplement: Supplementary file 1 — Supplementary Materials [file 41419_2024_6431_MOESM1_ESM.docx]

Supplementary Materials for

**Phosphorylation of INF2 by AMPK Promotes Mitochondrial Fission and Oncogenic Function in Endometrial Cancer**

**This file includes:**

Supplementary Figure 1 to 5

**Supplementary Figure 1.** INF2 expression is significantly up-regulated in EC (related to Figure 1).

**Supplementary Figure 2.** INF2 promotes EC cell proliferation partly by regulating mitochondrial dynamics (related to Figure 2).

**Supplementary Figure 3.** Validation of INF2 knockout in EC cell lines.

**Supplementary Figure 4.** AMPK interacts with INF2 and phosphorylates INF2 at Ser1077 (related to Figure 3, 4).

**Supplementary Figure 5.** AMPK-mediated phosphorylation of INF2 at Ser1077 promotes EC cell proliferation (related to Figure 5).

**Other Supplementary Materials for this manuscript include the following:**

Supplementary Table 1 to 4

**Supplementary Table 1.** IHC scores in 132 cases of endometrial cancer specimens and the associated clinical information.

**Supplementary Table 2.** Chemicals, recombinant proteins and antibodies.

**Supplementary Table 3.** SgRNA sequence information.

**Supplementary Table 4.** Recombinant DNA.

**
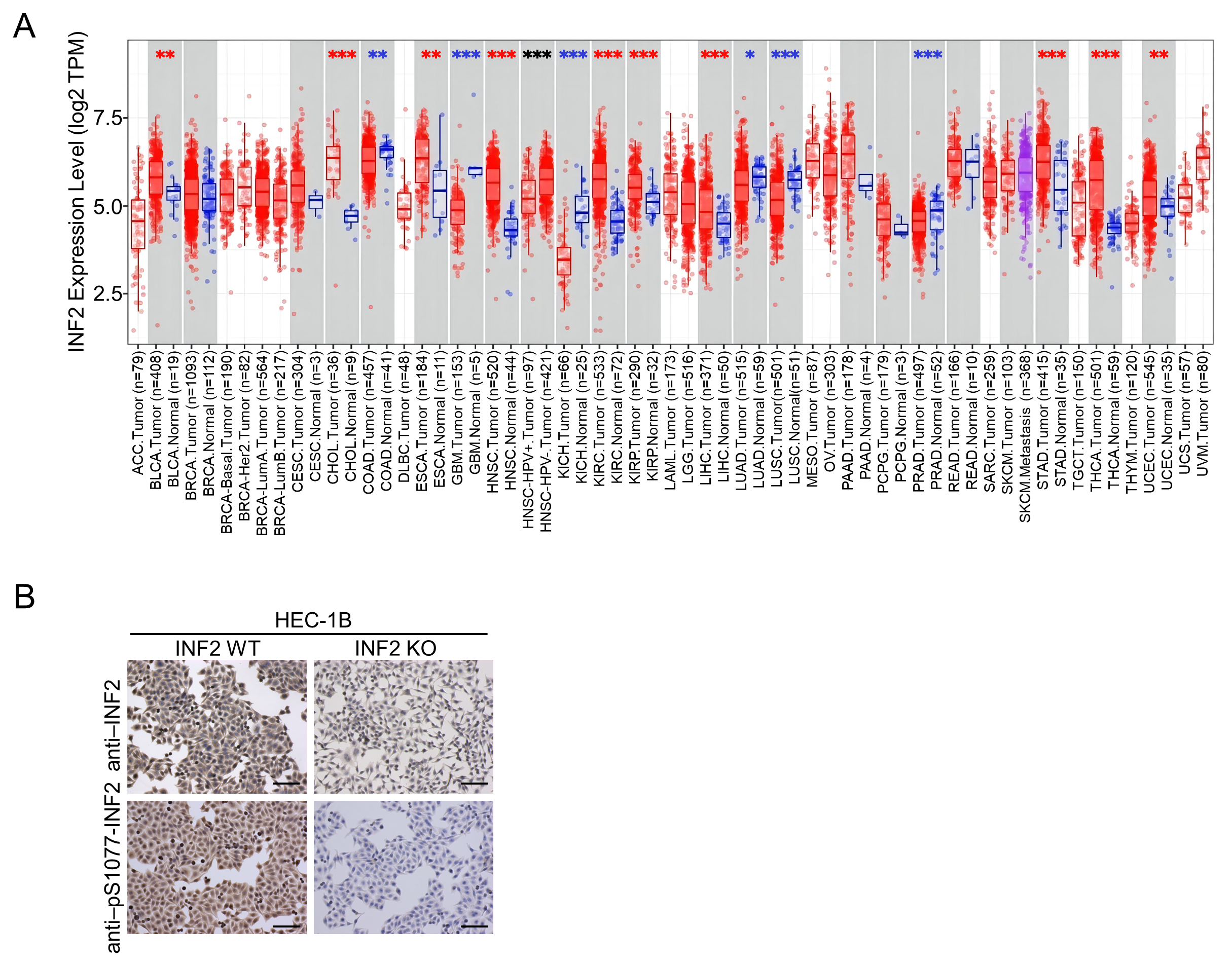
Supplementary Figure Legends**

**Supplementary Figure 1.** **INF2 expression is significantly up-regulated in EC (related to Figure 1).**

**(A)** Relative mRNA level of INF2 expression in pan-cancers from TCGA datasets (http://timer.cistrome.org/);

**(B)** Representative images of IHC staining in parental and INF2-KO HEC-1B cells with INF2 or INF2 (phospho-Ser1077) antibody. Scale bar,100 μm.


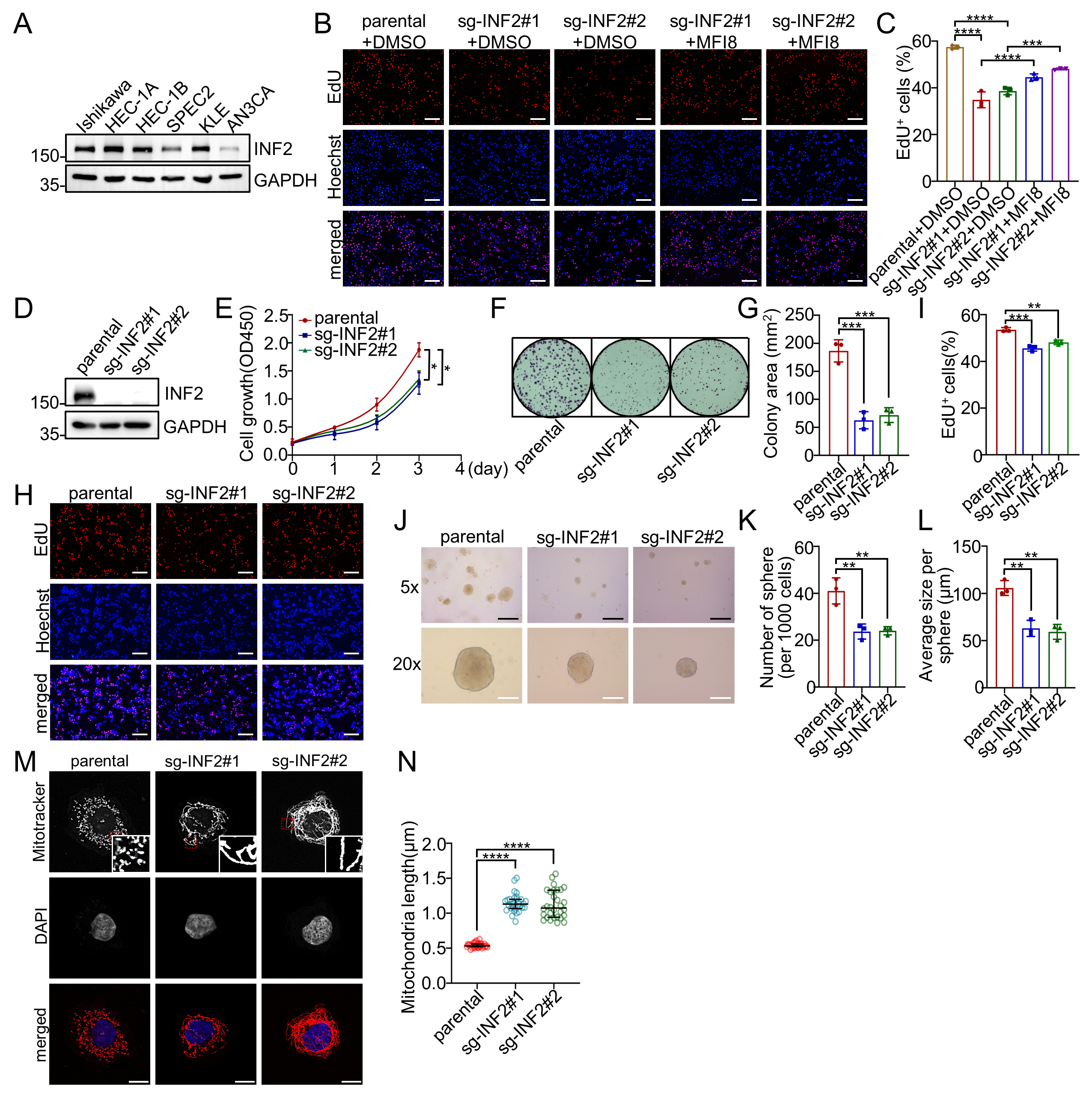
**Supplementary Figure 2.** **INF2 promotes EC cell proliferation partly by regulating mitochondrial dynamics (related to Figure 2).**

**(A)** WB analysis of INF2 protein levels in WCLs from a panel of EC cell lines.

**(B, C)** EdU assays were performed in INF2-KO HEC-1B cells treated with DMSO or MFI8 (20 μM) for 6 h, scalebar: 100μm. The proportion of EdU^+^ positive cells was analyzed statistically and shown in **(C)**. Data are shown as means ± SD (n = 30).

**(D)** INF2 KO Ishikawa cells were generated through LentiCRISPRv2 methods. The WCLs from parental and INF2 KO Ishikawa cells were prepared for WB with the indicated antibodies.

**(E)** CCK-8 assays were performed in parental and INF2-KO Ishikawa cells. Data are shown as means ± SD (n = 3).

**(F, G)** Colony formation assays were performed in parental and INF2-KO Ishikawa cells. The area of clones in **(F)** was analyzed statistically and shown in **(G)**. Data are shown as means ± SD (n = 3).

**(H, I)** EdU assays were performed in INF2-KO Ishikawa cells. The proportion of EdU^+^ positive cells was analyzed statistically and shown in (**I**). Data are shown as means ± SD (n = 3).

**(J-L)** 3D sphere formation assays were performed in parental and INF2-KO Ishikawa cells, scale bar: 5 ×, 200 μm; 20 ×, 50 μm. The numbers and sizes of cell spheres were analyzed statistically and shown in **(K, L)**. Data are shown as means ± SD (n = 3).

**(M, N)** Parental and INF2-KO Ishikawa cells were stained with DAPI and Mitotracker Orange. Representative confocal images are shown. Scale bar: 10 μm. The mitochondrial lengths were analyzed statistically and shown in **(N)**. Data are medians ± interquartile range (n = 30).

P values are calculated using the One-way ANOVA test in **(C, G, I, K, L)**, the Two-way ANOVA test in **(E)**, and the Kruskal-Wallis test in **(N)**. *p<0.05, **p<0.01, ***p<0.001, ****p<0.0001.

**
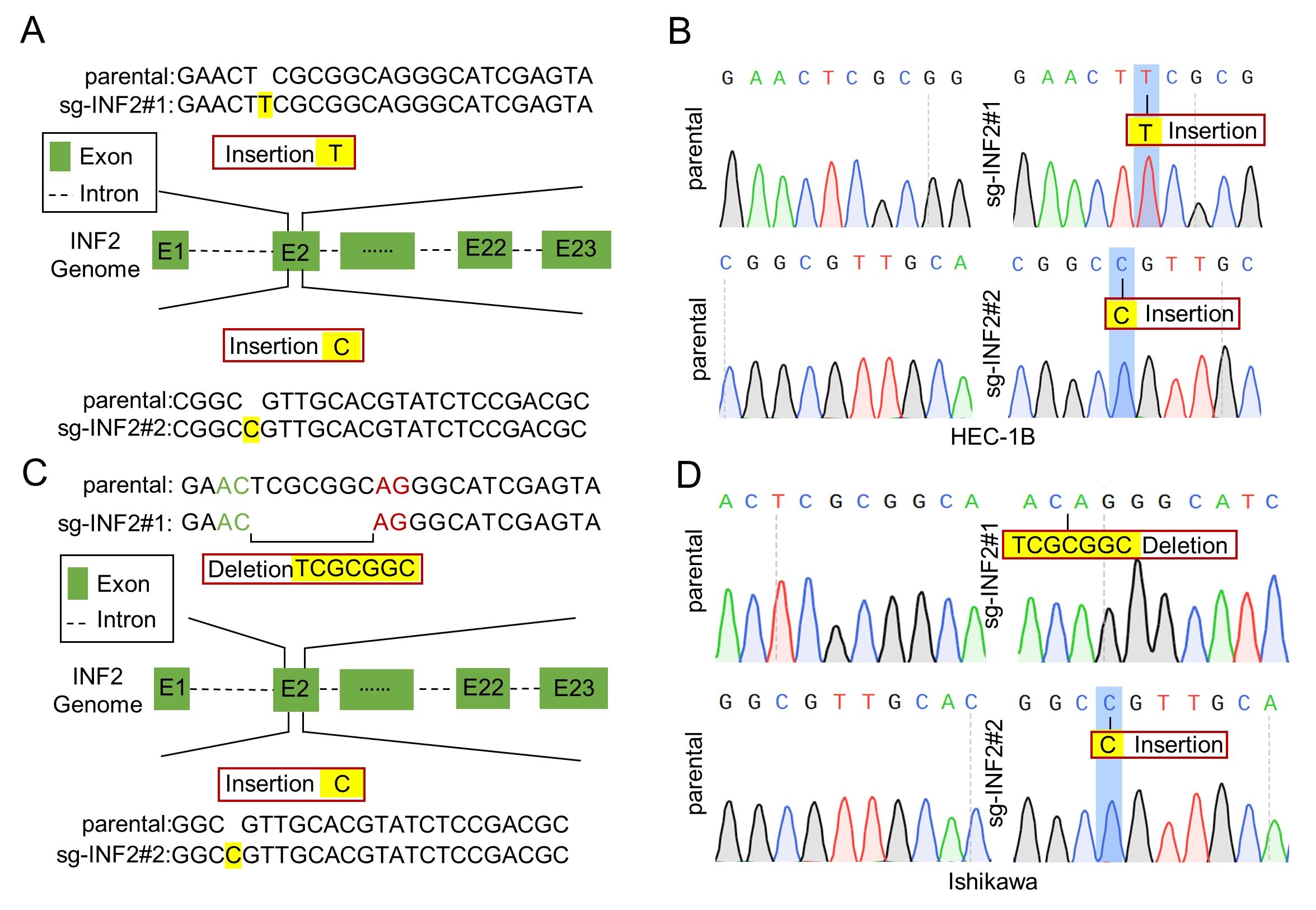
Supplementary Figure 3. Validation of INF2 knockout in EC cell lines.**

**(A, B)** Schematic of CRISPR/Cas9-mediated INF2 KO in HEC-1B cells and Sanger sequencing confirmed that the INF2 gene was edited by sgRNA#1 or sgRNA#2 in HEC-1B cells.

**(C, D)** Schematic of CRISPR/Cas9-mediated INF2 KO in Ishikawa cells and Sanger sequencing confirmed that the INF2 gene was edited by sgRNA#1 or sgRNA#2 in Ishikawa cells.


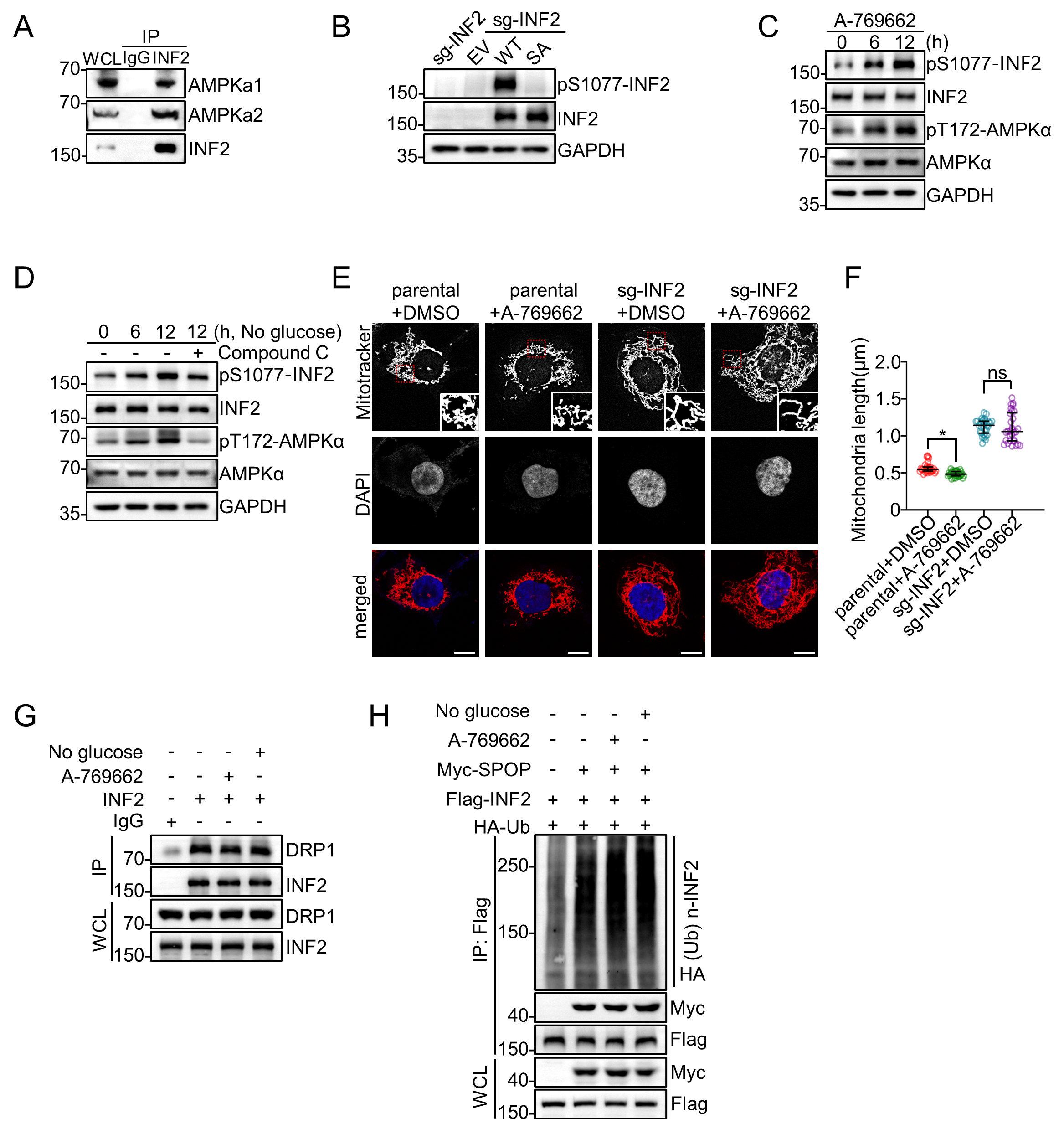
**Supplementary Figure 4.** **AMPK interacts with INF2 and phosphorylates INF2 at Ser1077 (related to Figure 3, 4).**

**(A)** Co-IP using anti-INF2 antibody in WCLs prepared from Ishikawa cells, followed by WB analysis with the indicated antibodies.

**(B)** Western blot of the Ishikawa cells transfected with indicated plasmids.

**(C)** WB analysis of the indicated proteins in WCLs from cells treated with A-769662 (100 μM) for the indicated times.

**(D)** WB analysis of the indicated proteins in WCLs from Ishikawa cells treated with glucose deprivation at the indicated times. The cells were pretreated with Compound C 2 h prior to glucose deprivation.

**(E, F)** Parental and INF2-KO Ishikawa cells were treated with DMSO or A-769662 (100 μM) for 6 h, then the cells were stained with DAPI and Mitotracker Orange. Representative confocal images are shown. Scale bar: 10 μm. The mitochondrial lengths were analyzed statistically and shown in **(F)**. Data are medians ± interquartile range (n = 30).

**(G)** Co-IP was performed using an anti-INF2 antibody on WCLs obtained from HEC-1B cells treated with DMSO, A-769662 (100 μM), or subjected to glucose deprivation for 12 h, followed by Western blot analysis with the specified antibodies.

**(H)** Western blot of the products of in vivo ubiquitination assays of 293T cells transfected with indicated plasmids and treated with DMSO, A-769662 (100 μM), or glucose deprivation for 12 h.

P values are calculated using the Kruskal-Wallis test in **(F)**. *p<0.05, ns: no significance.


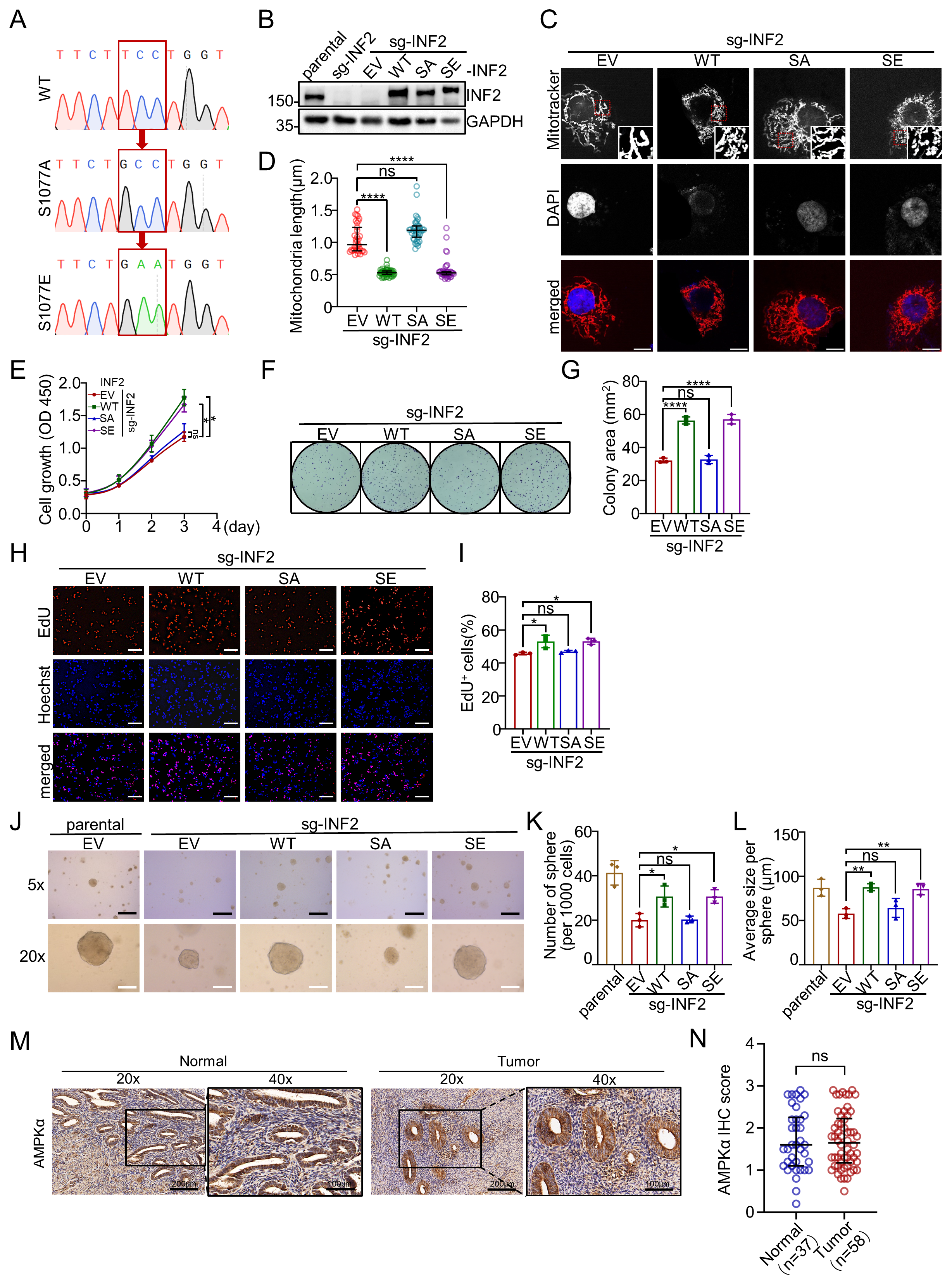
**Supplementary Figure 5.** **AMPK-mediated phosphorylation of INF2 at Ser1077 promotes EC cell proliferation (related to Figure 5).**

**(A)** The representative Sanger sequencing data of INF2-WT, SA, and SE constructs.

**(B)** WB analysis of the indicated proteins in WCLs from parental or INF2-KO Ishikawa cells stably expressing EV, INF2-WT, SA, or SE mutant.

**(C, D)** INF2-KO Ishikawa cells stably expressing EV, INF2-WT, SA, or SE mutant were stained with DAPI and Mitotracker Orange. Representative confocal images are shown in (**C**). Scale bar: 10 μm. The mitochondrial lengths were analyzed statistically and shown in (**D**). Data are medians ± interquartile range (n = 30).

**(E)** CCK-8 assays were performed in parental and INF2-KO Ishikawa cells. Data are shown as means ± SD (n = 3).

**(F, G)** Colony formation assays were performed in INF2-KO Ishikawa cells stably expressing EV, INF2-WT, SA, or SE mutant. The area of clones in **(F)** was analyzed statistically and shown in **(G)**. Data are shown as means ± SD (n = 3).

**(H, I)** EdU assays were performed in INF2-KO Ishikawa cells stably expressing EV, INF2-WT, SA, or SE mutant. The proportion of EdU^+^ positive cells was analyzed statistically and shown in **(I)**. Data are shown as means ± SD (n = 3).

**(J-L)** 3D sphere formation assays were performed in parental and INF2-KO Ishikawa cells, scale bar: 5 ×, 200 μm; 20 ×, 50 μm. The numbers and sizes of cell spheres were analyzed statistically and shown in **(K, L)**. Data are shown as means ± SD (n = 3).

**(M)** Representative IHC images of AMPKα staining in normal endometrium and EC tissues;

**(N)** Statistical analysis of AMPKα protein expression in (M). Data are medians ± interquartile range.

P values are calculated using the Kruskal-Wallis test in **(D)**, the Two-way ANOVA test in **(E)**, the One-way ANOVA test in **(G, I, K, L),** and the Mann-Whitney test in **(N)**. *p<0.05, **p<0.01, ****p<0.0001, ns: no significant.

**Supplementary Tables**

| **Sample** | **Pathology number** | **pT172-AMPK IHC score** | **pS1077-INF2 IHC score** | **INF2 IHC score** | **AMPKα IHC score** | **Histology** | **Clinical stage** |
| --- | --- | --- | --- | --- | --- | --- | --- |
| 1 | E2022-07798-06344 |  |  | 0.7 | 2.8 | Endometrium |  |
| 2 | 2022-13690-13017 |  |  | 0.2 | 1.2 | Endometrium |  |
| 3 | 2022-06223 |  |  | 1.6 | 2.2 | Endometrium |  |
| 4 | E2022-21282 |  |  | 1.6 | 1.6 | Endometrium |  |
| 5 | 2022-13498 |  |  | 1.1 | 2.7 | Endometrium |  |
| 6 | 2022-12054-11319 |  |  | 0.9 | 2 | Endometrium |  |
| 7 | E2021-24467 |  |  | 1.65 | 2.5 | Endometrium |  |
| 8 | 2022-12500 |  |  | 1.9 | 2.6 | Endometrium |  |
| 9 | 2022-12609-10957 |  |  | 1.8 | 1.1 | Endometrium |  |
| 10 | E2021-37654 |  |  | 1.8 | 2.3 | Endometrium |  |
| 11 | E2021-27771 |  |  | 1 | 1 | Endometrium |  |
| 12 | 2023-01010-00529 |  |  | 0.4 | 1.1 | Endometrium |  |
| 13 | 2022-13599 |  |  | 1.75 | 0.8 | Endometrium |  |
| 14 | 2022-09751-10768 |  |  | 1.9 | 1.5 | Endometrium |  |
| 15 | E2022-21134-19722 |  |  | 0.5 | 1 | Endometrium |  |
| 16 | 2022-14074 |  |  | 1.6 | 2.8 | Endometrium |  |
| 17 | 2022-10153 |  |  | 1.9 |  | Endometrium |  |
| 18 | E2021-21087-19069 |  |  | 1.5 | 1 | Endometrium |  |
| 19 | 2022-11347-10256 |  |  | 1.9 | 0.2 | Endometrium |  |
| 20 | 19ZGP044-PP2 |  |  |  | 1.5 | Endometrium |  |
| 21 | 19ZGP038-PP1 |  |  |  | 2.8 | Endometrium |  |
| 22 | 21ZGP011-PP1 |  |  |  | 1.2 | Endometrium |  |
| 23 | 21ZGP008-PP1 |  |  |  | 2 | Endometrium |  |
| 24 | 18ZGP057-PP1 |  |  |  | 1.6 | Endometrium |  |
| 25 | 21ZGP118-PP1 |  |  |  | 0.5 | Endometrium |  |
| 26 | 21ZGP016-PP1 |  |  |  | 2 | Endometrium |  |
| 27 | 21ZGP028-PP1 |  |  |  | 2.9 | Endometrium |  |
| 28 | 21ZGP104-PP1 |  |  |  | 2.8 | Endometrium |  |
| 29 | 19ZGP052-PP1 |  |  |  | 2.2 | Endometrium |  |
| 30 | 19ZGP093-PP2 |  |  |  | 1 | Endometrium |  |
| 31 | 21ZGP161-PP2 |  |  |  | 1.5 | Endometrium |  |
| 32 | 21ZGP158-PP1 |  |  |  | 1.6 | Endometrium |  |
| 33 | 21ZGP136-NP1 |  |  |  | 1.2 | Endometrium |  |
| 34 | 21ZGP135-NP1 |  |  |  | 1 | Endometrium |  |
| 35 | 21ZGP139-NP1 |  |  |  | 1.3 | Endometrium |  |
| 36 | 21ZGP138-NP1 |  |  |  | 2.2 | Endometrium |  |
| 37 | 21ZGP137-NP1 |  |  |  | 1.7 | Endometrium |  |
| 38 | 21ZGP134-NP1 |  |  |  | 1.4 | Endometrium |  |
| 39 | 17-ZGP013b | 0.7 | 0.7 | 1 | 2.1 | Endometrioid | IA |
| 40 | 16-ZGP036-E | 0.1 | 1.2 | 0.9 | 1.6 | Endometrioid | IA |
| 41 | E2016-18516-3 | 1.1 | 1.9 | 1.6 | 1.9 | Endometrioid | IA |
| 42 | E2016-06049-2 | 0.5 | 2.3 | 0.9 | 1.6 | Endometrioid | IA |
| 43 | 16-ZGP-032f | 1.4 | 1 | 1 | 1.8 | Endometrioid | IA |
| 44 | E2016-01748-1 | 0.6 | 0.8 | 0.5 | 1.7 | Endometrioid | IA |
| 45 | 16-ZGP045-d | 0.7 | 2.7 | 2.7 | 1.3 | Endometrioid | IIIC |
| 46 | 17-ZGP065-b | 0.2 | 0.4 | 1 | 1.1 | Endometrioid | IA |
| 47 | E2016-12519-8 | 1 | 1.6 | 1.2 | 1.3 | Endometrioid | IA |
| 48 | 17ZGP005e | 0.7 | 2.2 | 1.6 | 2.3 | Endometrioid | IA |
| 49 | E2014-05215-3 | 0.05 | 0.3 | 0.9 | 1.1 | Endometrioid | IA |
| 50 | 2016-10509-2 | 2.3 | 2.7 | 2.85 |  | Endometrioid | IA |
| 51 | E2014-03005-1 | 0.05 | 0.2 | 2.6 |  | Endometrioid | IB |
| 52 | 15-ZGP057-f | 0.01 | 0.05 | 2.1 |  | Endometrioid | IB |
| 53 | E2016-12145-1 | 0.8 | 0.7 | 0.9 |  | Endometrioid | IA |
| 54 | E2016-22231-2 | 0.02 | 0.05 | 0.5 |  | Endometrioid | IA |
| 55 | 18-ZGP-003d | 0.2 | 0.3 | 1.2 |  | Endometrioid | IA |
| 56 | 13-7850-3 | 0.5 | 1.1 | 1.8 |  | Endometrioid | IA |
| 57 | 17-ZGP021-B | 1 | 1.15 | 1 | 2.4 | Endometrioid | IA |
| 58 | E2016-01763-4E | 0.02 | 0.05 | 0.9 | 0.95 | Endometrioid | IA |
| 59 | 15-ZGP046d | 0.1 | 0.1 | 1.5 | 1.5 | Endometrioid | IB |
| 60 | 17-ZGP046-b | 0.2 | 0.05 | 0.8 | 1.8 | Endometrioid | IA |
| 61 | 17-ZGP-002e | 0.05 | 0.02 | 1.9 | 2 | Endometrioid | IB |
| 62 | 16-ZGP-034b | 0.8 | 0.01 | 0.9 | 2.8 | Endometrioid | IA |
| 63 | E2016-05350-4 | 1.1 | 0.8 | 1 | 1.4 | Endometrioid | IB |
| 64 | 2016-13985-8 | 0.7 | 0.1 | 3 | 2.8 | Endometrioid | IA |
| 65 | E2016-00379-1 | 0.4 | 0.3 | 0.3 | 1 | Endometrioid | IA |
| 66 | 17-ZGP-029d | 2.6 | 2.9 | 2.4 |  | Mix | IA |
| 67 | 2014-11279-1 | 1 | 1.9 | 2.5 |  | Endometrioid | IA |
| 68 | 11-25161-4 | 2.4 | 2.8 | 2.35 |  | Endometrioid | IA |
| 69 | 15-ZGP-001b | 0.1 | 0.7 | 1.5 |  | Endometrioid | IA |
| 70 | 10-15697-F | 1.4 | 1.45 | 1.6 |  | Endometrioid | IA |
| 71 | 13-19518-6 | 1.4 | 1.3 | 2 |  | Endometrioid | IA |
| 72 | 11-01269 | 1.4 | 2 | 1.9 |  | Endometrioid | IA |
| 73 | 15-ZGP-039c | 2.8 | 2.6 | 2.6 |  | Endometrioid | IA |
| 74 | 15-ZGP-031m | 2.1 | 2.3 | 2.8 |  | Endometrioid | II |
| 75 | E2014-05690-1 | 2.25 | 2.45 | 2.8 |  | Endometrioid | IB |
| 76 | 13-20543-5 | 1.6 | 2 | 2.6 |  | Endometrioid | IB |
| 77 | 13-4425-6 | 0.8 | 0.9 | 1.6 |  | Endometrioid | IA |
| 78 | E2022-07798-06344 | 0.7 | 1.5 | 2 | 1.3 | Endometrioid | IIIC |
| 79 | 2022-13690-13017 | 2.7 | 2.7 | 3 | 1.05 | Endometrioid | IA |
| 80 | 2022-06223 | 1.4 | 1.3 | 1.8 |  | Serous | IA |
| 81 | E2022-21282 | 0.3 | 1.95 | 1.5 | 0.8 | Endometrioid | IIIC |
| 82 | 2022-13498 | 1.1 | 2.1 | 2.6 |  | Serous | IIIC |
| 83 | 2022-12054-11319 | 1.8 | 2 | 1.3 |  | Endometrioid | II |
| 84 | E2021-24467 | 1.6 | 2.65 | 2.5 | 1.8 | Endometrioid | II |
| 85 | 2022-12500 | 1.3 | 2.8 | 2.9 |  | Endometrioid | IIIC |
| 86 | 2022-12609-10957 | 1.5 | 2.2 | 2.2 |  | Endometrioid | IA |
| 87 | E2021-37654 | 0.9 | 2.8 | 0.5 | 1 | Endometrioid | IA |
| 88 | E2021-27771 | 0.5 | 2.7 | 2.7 | 1.3 | Mix | IIIC |
| 89 | 2023-01010-00529 | 2.7 | 2.8 | 2.9 | 1.2 | Endometrioid | IA |
| 90 | 2022-13599 | 1.9 | 2.65 | 2.65 | 1.8 | Serous | IA |
| 91 | 2022-09751-10768 | 2.1 | 2.4 | 2.7 | 1.1 | Endometrioid | IA |
| 92 | E2022-21134-19722 | 2.7 | 1.9 | 2.4 | 0.5 | Serous | IIIC |
| 93 | 2022-14074 | 0.1 | 2.7 | 2.7 | 0.8 | Endometrioid | IIIC |
| 94 | 2022-10153 | 0.75 | 2.9 | 3 |  | Serous | II |
| 95 | E2021-21087-19069 | 1.5 | 2.85 | 2.95 | 1.9 | Serous | IA |
| 96 | 2022-11347-10256 | 2.3 | 2.2 | 2.2 |  | Endometrioid | IA |
| 97 | 13-11504-3 | 1.1 | 2 |  | 0.8 | Serous | IA |
| 98 | 2015-00893 | 2.6 | 2.9 |  | 2.3 | Serous | IA |
| 99 | E2014-07219-2 | 0.1 | 0.5 |  |  | Serous | IIIC |
| 100 | 16-ZGP-039e | 2 | 1.5 |  | 2.1 | Endometrioid | IA |
| 101 | 17-ZGP032-g | 0.05 | 0.1 |  | 2.5 | Carcinosarcomas | IV |
| 102 | 2015-03808-2 | 0.02 | 0.1 |  | 1.2 | Serous | IA |
| 103 | 10-21285-2 | 0.2 | 0.1 |  | 2.2 | Clear cell carcinoma | IIIB |
| 104 | E2016-05842-6 | 0.2 | 0.1 |  | 1 | Serous | IA |
| 105 | 13-20800-3 | 0.8 | 0.3 |  | 1.3 | Serous | IA |
| 106 | 17-ZGP-071c | 2.1 | 2.2 |  |  | Carcinosarcomas | IB |
| 107 | 11-20422-5 | 0.6 | 0.7 |  |  | Endometrioid | IA |
| 108 | 15-ZGP-032h | 1.55 | 1.5 |  |  | Endometrioid | IB |
| 109 | 15-ZGP-028d | 2.5 | 2.7 |  |  | Endometrioid | II |
| 110 | E2014-05625-8 |  |  | 1.5 |  | Endometrioid | IA |
| 111 | 15-ZGP-051c |  |  | 2.1 |  | Endometrioid | IA |
| 112 | 13-5169 |  |  | 1.3 |  | Endometrioid | IA |
| 113 | 11-03407 |  |  | 2.6 |  | Endometrioid | II |
| 114 | 13-4762-4 |  |  | 1.3 |  | Endometrioid | IA |
| 115 | 21ZGP104-TP2 |  |  |  | 2.8 | Endometrioid | I |
| 116 | 21ZGP158-TP1 |  |  |  | 1.4 | Endometrioid | I |
| 117 | 21ZGP028-TP1 |  |  |  | 2 | Endometrioid | II |
| 118 | 19ZGP052-TP1 |  |  |  | 1.4 | Serous | III |
| 119 | 21ZGP057-TP2 |  |  |  | 2.9 | Serous | I |
| 120 | 19ZGP038-TP1 |  |  |  | 2.8 | Endometrioid | I |
| 121 | 21ZGP057-TP3 |  |  |  | 1 | Serous | I |
| 122 | 21ZGP011-TP1 |  |  |  | 1.9 | Endometrioid | II |
| 123 | 21ZGP008-TP1 |  |  |  | 1.7 | Endometrioid | I |
| 124 | 21ZGP030-TP3 |  |  |  | 1.2 | Endometrioid | III |
| 125 | 19ZGP093-TP1 |  |  |  | 0.9 | Endometrioid | II |
| 126 | 21ZGP118-TP1 |  |  |  | 2.8 | Endometrioid | I |
| 127 | 21ZGP016-TP1 |  |  |  | 2.9 | Serous | I |
| 128 | 21ZGP062-TP1 |  |  |  | 2.85 | Endometrioid | I |
| 129 | 19ZGP044-TP1 |  |  |  | 1.3 | Endometrioid | II |
| 130 | 19ZGP032-TP4 |  |  |  | 2.4 | Endometrioid | III |
| 131 | 18ZGP057-TP2 |  |  |  | 2.4 | Endometrioid | I |
| 132 | 21ZGP161-TP1 |  |  |  | 1.9 | Endometrioid | I |

**Supplementary Table 1. IHC scores in 132 cases of endometrial cancer specimens and the associated clinical information.**

| **No** | **Reagent** | **Source** | **Cat. No** | |
| --- | --- | --- | --- | --- |
| 1 | DMEM | BI | Cat# C3113-0500 | |
| 2 | DMEM/F12 | BI | Cat# C3130-0500 | |
| 3 | Fetal Bovine Serum | BI | Cat# 04-001-1A | |
| 4 | Penicillin-Streptomycin | Thermo Fisher | Cat# 15140122 | |
| 5 | Puromycin | Thermo Fisher | Cat# A1113803 | |
| 6 | DMEM (no glucose) | Gibco | Cat# 11966025 | |
| 7 | Protease Inhibitor Cocktail | Thermo Fisher | Cat# 78441 | |
| 8 | Minute(TM) ER Enrichment Kit For Tissues and Cultured Cells | Invent | Cat# ER-036 | |
| 9 | ECL system | Millipore | Cat# WBULS0100 | |
| 10 | EdU Cell Proliferation Kit | Beyotime | Cat# C0075 | |
| 11 | Lipofectamine 2000 | Thermo Fisher | Cat# 11668027 | |
| 12 | DMEM/F12 (no glucose) | Procell | Cat# PM150322 | |
| 13 | [Phanta Max Super-Fidelity DNA Polymerase](http://www.vazymebiotech.com/products_detail/productId=119.html) | Vazyme Biotech | Cat# P505 | |
| 14 | CCK-8 | MCE | Cat# HY-K0301 | |
| 15 | Matrigel | Corning | Cat# 356234 | |
| 16 | Protein A/G-conjugated Agarose Beads | Abmart | Cat# A10001 | |
| 17 | Compound C | Merck | Cat# 171260 | |
| 18 | A-769662 | APExBIO | Cat# A3963 | |
| 19 | AMPKα1/β1/γ1 | Carna Biosciences | Cat# 02-413-20N | |
| 20 | MFI8 | MCE | Cat# 694488-83-0 | |
| 21 | DMSO | Sigma | Cat# D2650 | |
| 22 | MG132 | Selleckchem | Cat# S2619 | |
| **No** | **Antibody/Cytokine** | **Source** | **Cat. No** | **Dilution** |
| 1 | GAPDH | proteintech | 60004-1-Ig | IB: 1:10000 |
| 2 | INF2 | proteintech | 20466-1-AP | IB: 1:2000 / IHC: 1:200 |
| 3 | DRP1 | CST | 8570S | IB: 1:1000 |
| 4 | AMPKα1 | proteintech | 10929-2-AP | IB: 1:1000 |
| 5 | AMPKα2 | proteintech | 18167-1-AP | IB: 1:1000 |
| 6 | Phospho-AMPKα (Thr172) | CST | 2535 | IB: 1:1000 / IHC: 1:20 |
| 7 | VDAC2 | Abclonal | A16294 | IB: 1:1000 |
| 8 | Calreticulin | Abcam | ab92516 | IB: 1:1000 |
| 9 | Myc | proteintech | 10828-1-AP | IB: 1:2000 |
| 10 | GST | CST | 2625S | IB: 1:1000 |
| 11 | Actin | CST | 3700S | IB: 1:1000 |
| 12 | Phospho-INF2 (S1077) | Abclonal | NA | IB: 1:2500 / IHC:1:50 |
| 13 | Flag | MBL | M185-7 | IB:1:5000 |
| 14 | Phospho-AMPK Substrate Motif LXRXX(pS/pT) | CST | 5759 | IB:1:1000 |
| 15 | AMPKα | CST | 2532S | IB: 1:1000 |
| 16 | His | CST | 2366S | IB: 1:1000 |
| 17 | Anti-rabbit | proteintech | SA00001-2 | IB: 1:2000 |
| 18 | Anti-mouse | CST | 7076S | IB: 1:2000 |
| IB:Immunoblot；IHC:Immuobiochemistry | | | | |

**Supplementary Table 2. Chemicals, recombinant proteins and antibodies.**

| **Gene** | **Sequence** |
| --- | --- |
| sg-INF2-1 | TGCGCGCCGTCATGAACTCG |
| sg-INF2-2 | CGGAGATACGTGCAACGCCG |

**Supplementary Table 3. SgRNA sequence information.**

| pCMV-Flag-N-INF2 | This Study | NM_022489.4 |
| --- | --- | --- |
| pCMV-Flag-N-INF2-S1077A | This Study | NA |
| pCMV-Flag-N-INF2-S1077E | This Study | NA |
| pCMV-Myc-C-INF2 | This Study | NM_022489.4 |
| pCDH-N-Flag-INF2 | This Study | NM_022489.4 |
| pCDH-N-Flag-INF2-S1077A | This Study | NA |
| pCDH-N-Flag-INF2-S1077E | This Study | NA |
| pcDNA3.1-N-FLAG-AMPKα1 | This Study | NM_006251.6 |
| pcDNA3.1-N-FLAG-AMPKα2 | This Study | NM_006252.4 |
| GST-INF2_900-1249aa_ | This Study | NA |
| GST-INF2_900-1249aa_-S1077A | This Study | NA |

**Supplementary Table 4. Recombinant DNA.**
