# Supplementary material for: Phosphorylation of INF2 by AMPK promotes mitochondrial fission and oncogenic function in endometrial cancer
Source: Cell Death Dis. 2024 Jan 17;15(1):65. doi: 10.1038/s41419-024-06431-0 (PMC10794193; doi:10.1038/s41419-024-06431-0)

Figure 2A

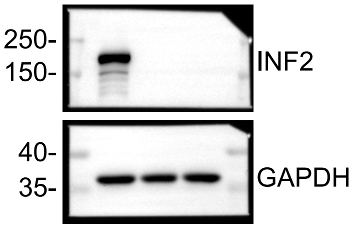


Figure 3A

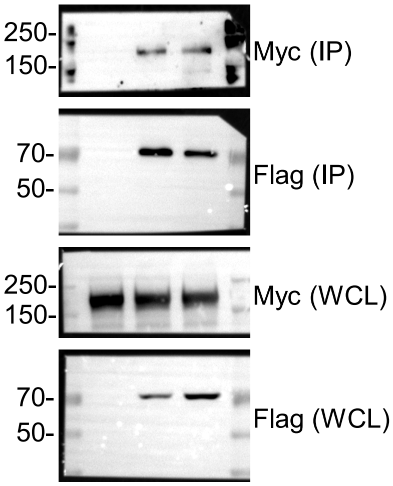


Figure 3B

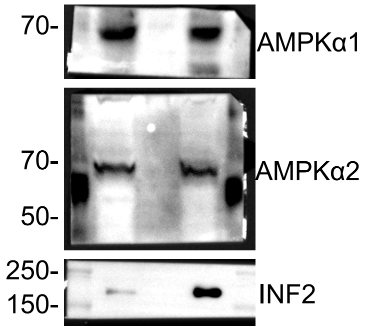


Figure 3C

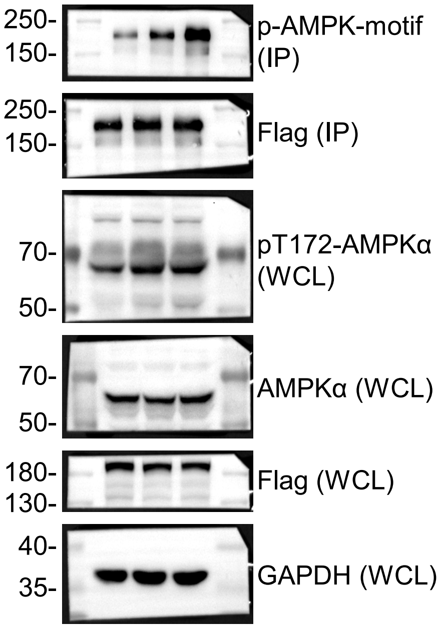


Figure 3E

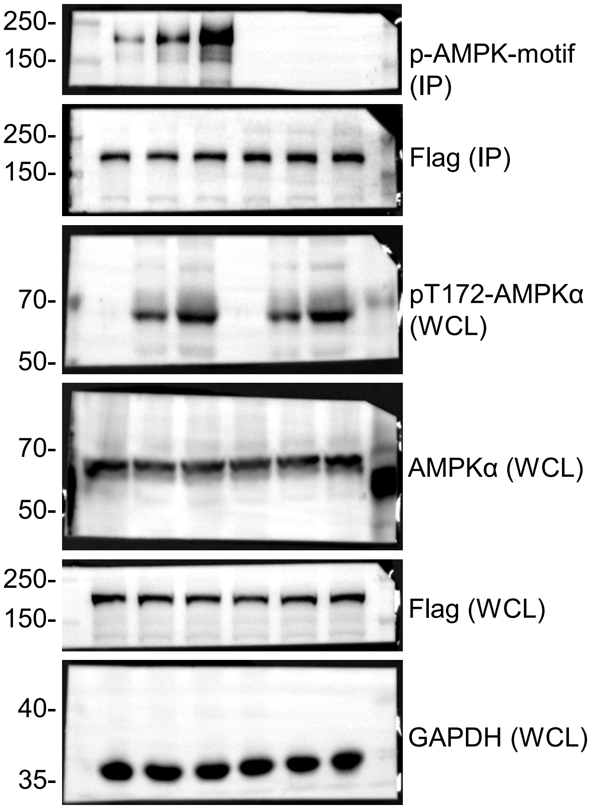

Figure 3F

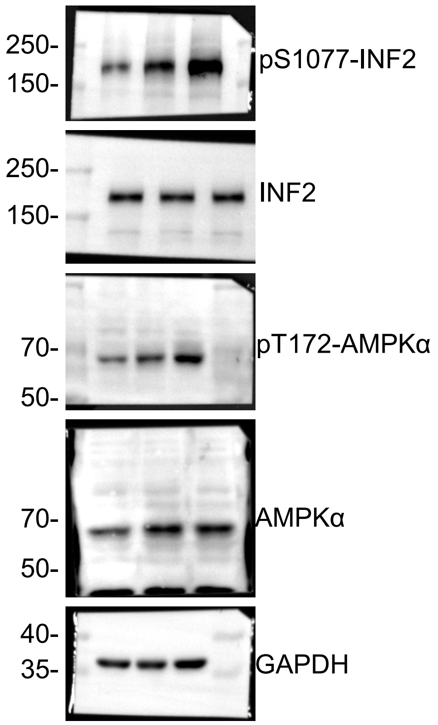


Figure 3G

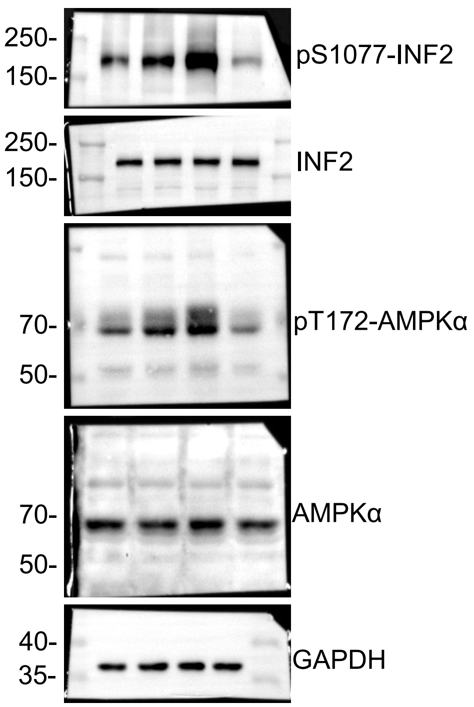


Figure 3H

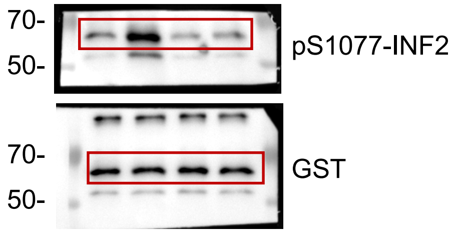

Figure 4E

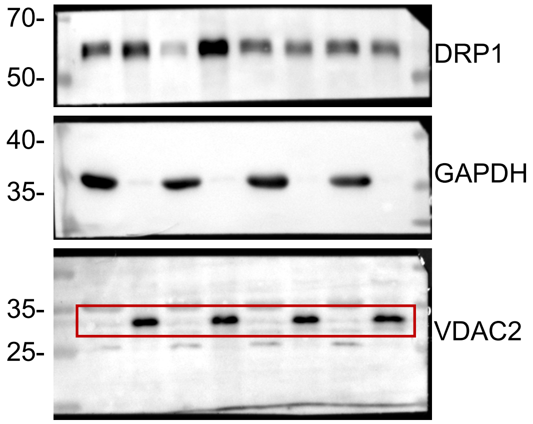


Figure 4F

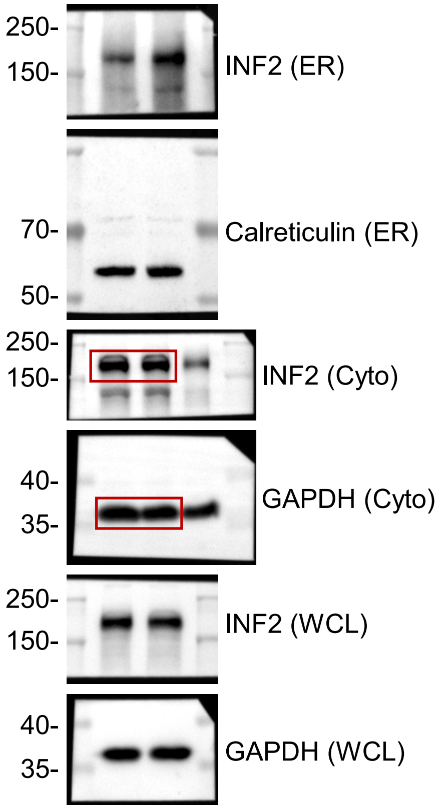


Figure 5A

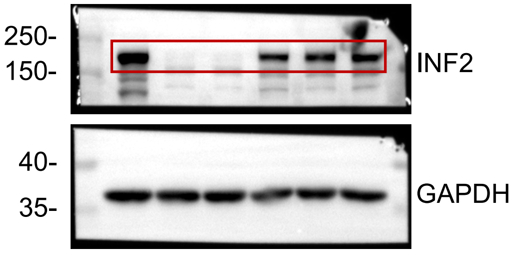


Figure 5F

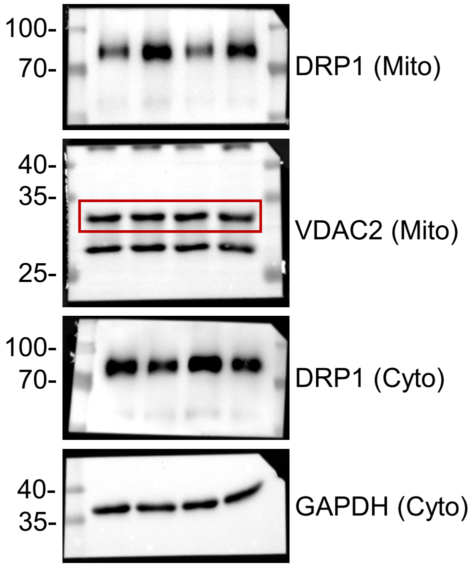

Supplementary Figure 2A

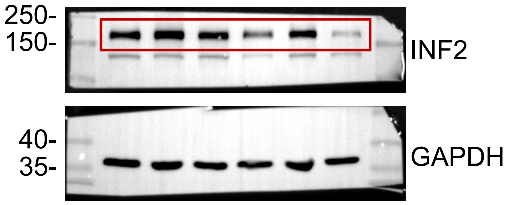


Supplementary Figure 2D

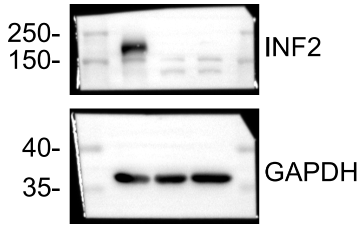


Supplementary Figure 4A

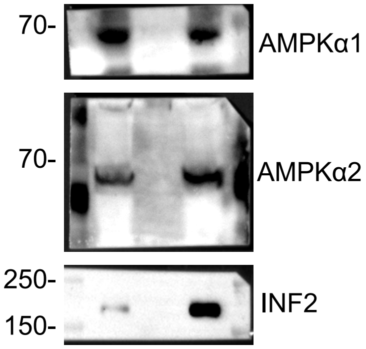


Supplementary Figure 4B

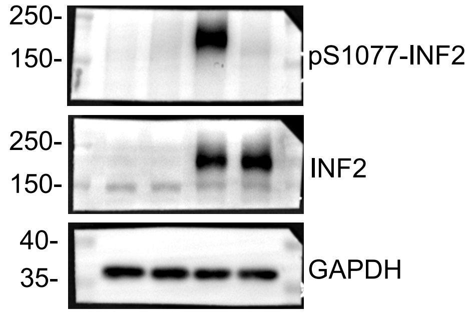


Supplementary Figure 4C

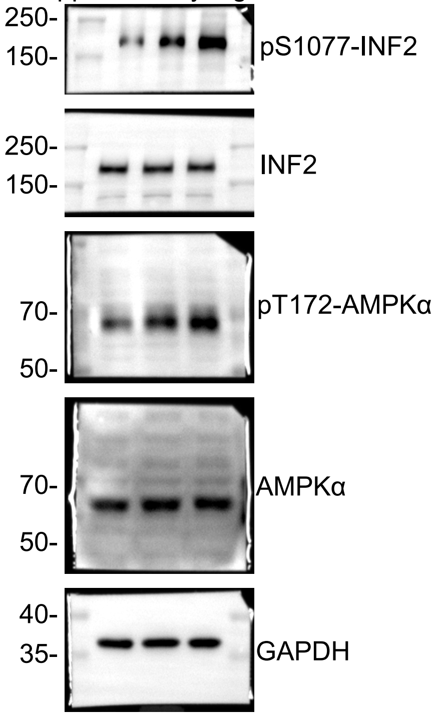


Supplementary Figure 4D

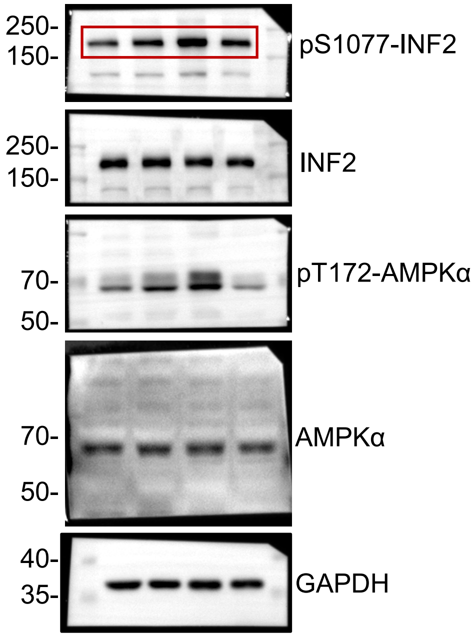


Supplementary Figure 4G

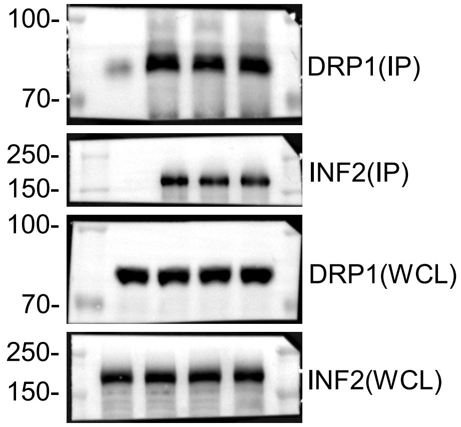


Supplementary Figure 4H

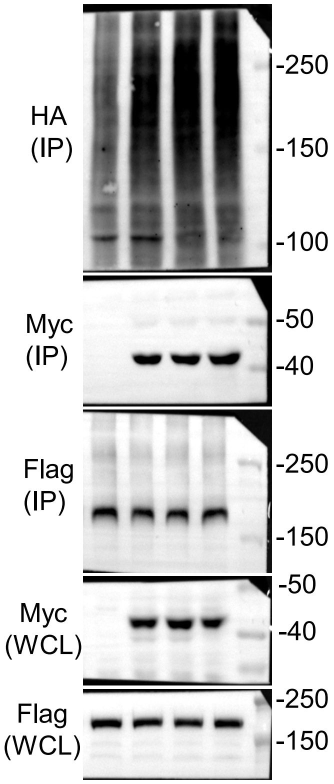


Supplementary Figure 5B

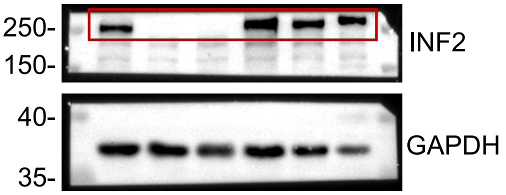

Supplement: Supplementary file 2 — Original Data File [file 41419_2024_6431_MOESM2_ESM.pdf]
